# Current progress on engineering microbial strains and consortia for production of cellulosic butanol through consolidated bioprocessing

**DOI:** 10.1111/1751-7915.14148

**Published:** 2022-09-27

**Authors:** Angela Re, Roberto Mazzoli

**Affiliations:** ^1^ Centre for Sustainable Future Technologies Fondazione Istituto Italiano di Tecnologia Torino Italy; ^2^ Department of Applied Science and Technology Politecnico di Torino Turin Italy; ^3^ Structural and Functional Biochemistry, Laboratory of Proteomics and Metabolic Engineering of Prokaryotes, Department of Life Sciences and Systems Biology University of Torino Torino Italy

## Abstract

In the last decades, fermentative production of n‐butanol has regained substantial interest mainly owing to its use as drop‐in‐fuel. The use of lignocellulose as an alternative to traditional acetone–butanol–ethanol fermentation feedstocks (starchy biomass and molasses) can significantly increase the economic competitiveness of biobutanol over production from non‐renewable sources (petroleum). However, the low cost of lignocellulose is offset by its high recalcitrance to biodegradation which generally requires chemical‐physical pre‐treatment and multiple bioreactor‐based processes. The development of consolidated processing (i.e., single‐pot fermentation) can dramatically reduce lignocellulose fermentation costs and promote its industrial application. Here, strategies for developing microbial strains and consortia that feature both efficient (hemi)cellulose depolymerization and butanol production will be depicted, that is, rational metabolic engineering of native (hemi)cellulolytic or native butanol‐producing or other suitable microorganisms; protoplast fusion of (hemi)cellulolytic and butanol‐producing strains; and co‐culture of (hemi)cellulolytic and butanol‐producing microbes. Irrespective of the fermentation feedstock, biobutanol production is inherently limited by the severe toxicity of this solvent that challenges process economic viability. Hence, an overview of strategies for developing butanol hypertolerant strains will be provided.

## Graphical abstract

Development of consolidated bioprocessing (CBP) of lignocellulose to butanol can potentially replace butanol production from non‐renewable sources at viable costs. Progress in CBP development has been obtained by combination of (hemi)cellulolytic and butanol‐producing features in a single strain (by metabolic engineering or protoplast fusion) or by artificial microbial consortia. Development of butanol hypertolerant strains is required to address inherent butanol toxicity and increase fermentation titer.

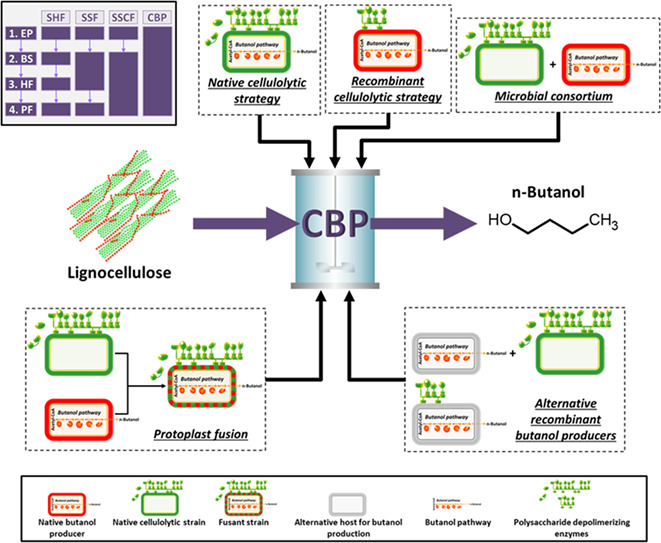

## INTRODUCTION

n‐Butanol (1‐butanol, hereinafter mentioned simply as butanol) has attracted substantial research interest in the last decades owing to its application as a drop‐in fuel in addition to its uses as a precursor of paints, polymers, and plastics (Gu et al., 2011; Jiang et al., 2015). With respect to ethanol, butanol has properties more similar to that of gasoline (high combustion energy, low volatility, and corrosivity) (Dürre, 2007), therefore, pure butanol can be fed to spark ignited engines without any modification (Campos‐Fernández et al., 2012).

The first industrial production of butanol was performed more than a century ago, through the so‐called ABE (that stands for acetone, butanol, and ethanol, in a 3:6:1 ratio) fermentation of starch or sugar by the solventogenic bacterium *Clostridium acetobutylicum* (Jones & Woods, 1986). Since the 1960s, bio‐based production of butanol has essentially been replaced by cheaper petroleum‐based processes with few exceptions (Green, 2011; Jiang et al., 2015). The high cost of feedstocks (it may represent over 70% of the total fermentation cost) and low solvent titer, yield, and productivity (corresponding to ≈20 g L^−1^, ≈0.33 g g^−1^, and <0.5 g L^−1^ h^−1^, respectively, as regards butanol) were among the factors limiting ABE process economics (Abo et al., 2019; Gu et al., 2011). More recently, biobutanol has regained considerable attention in the perspective of enhancing process environmental sustainability (Azambuja & Goldbeck, 2020; Bao et al., 2020; Ferreira et al., 2020; Li et al., 2021; Nawab et al., 2020; Wen, Li, Liu, Jin, & Yang, 2020). In addition to ABE fermentation, processes based on native or engineered microorganisms that produce isopropanol–butanol–ethanol (IBE) mixtures, although less efficient, are currently investigated since these solvent mixtures are potential automotive fuels (Cui et al., 2020a, 2020b; dos Santos Vieira et al., 2019). Promising new generation feedstocks for butanol fermentation include food wastes because of their large accumulation and high starch content (Qin et al., 2018; Su et al., 2022; Zhang et al., 2020). Lignocellulosic biomass (which also includes many agricultural, municipal, and industrial wastes) is an alternative abundant and inexpensive fermentation feedstock (Sims et al., 2010). As an example, no cost can be attributed to food waste, the current price of pulp‐grade wood can be estimated at 43–54 US$/ton of fermentable sugars (i.e., cellulose and hemicellulose) while sugar costs about 460 US$/ton (Gharehkhani et al., 2015; International Sugar Organization, 2019; Nuss & Gardner, 2013; Qureshi et al., 2020). Recent techno‐economic analyses estimated the minimal selling price for butanol produced from the fermentation of corn, sugarcane, food/municipal waste, and lignocellulosic biomass at 2.50, 2.05, 0.42–0.75, and 1.32–1.78 US$/kg, respectively (Ashani et al., 2020; Karimi Alavijeh & Karimi, 2019; Mailaram & Maity, 2022; Qureshi et al., 2020). These values may be largely affected by fluctuations in feedstock price, process configuration, plant capacity, and location. However, these estimates highlight the current potential of biobutanol to compete with petroleum‐derived butanol (whose price has recently increased to 1.72–2.87 US$/kg) (“N‐Butanol (NBA) Pricing, Prices, Price, Demand & Supply | ChemAnalyst,” 2022).

**FIGURE 1:**
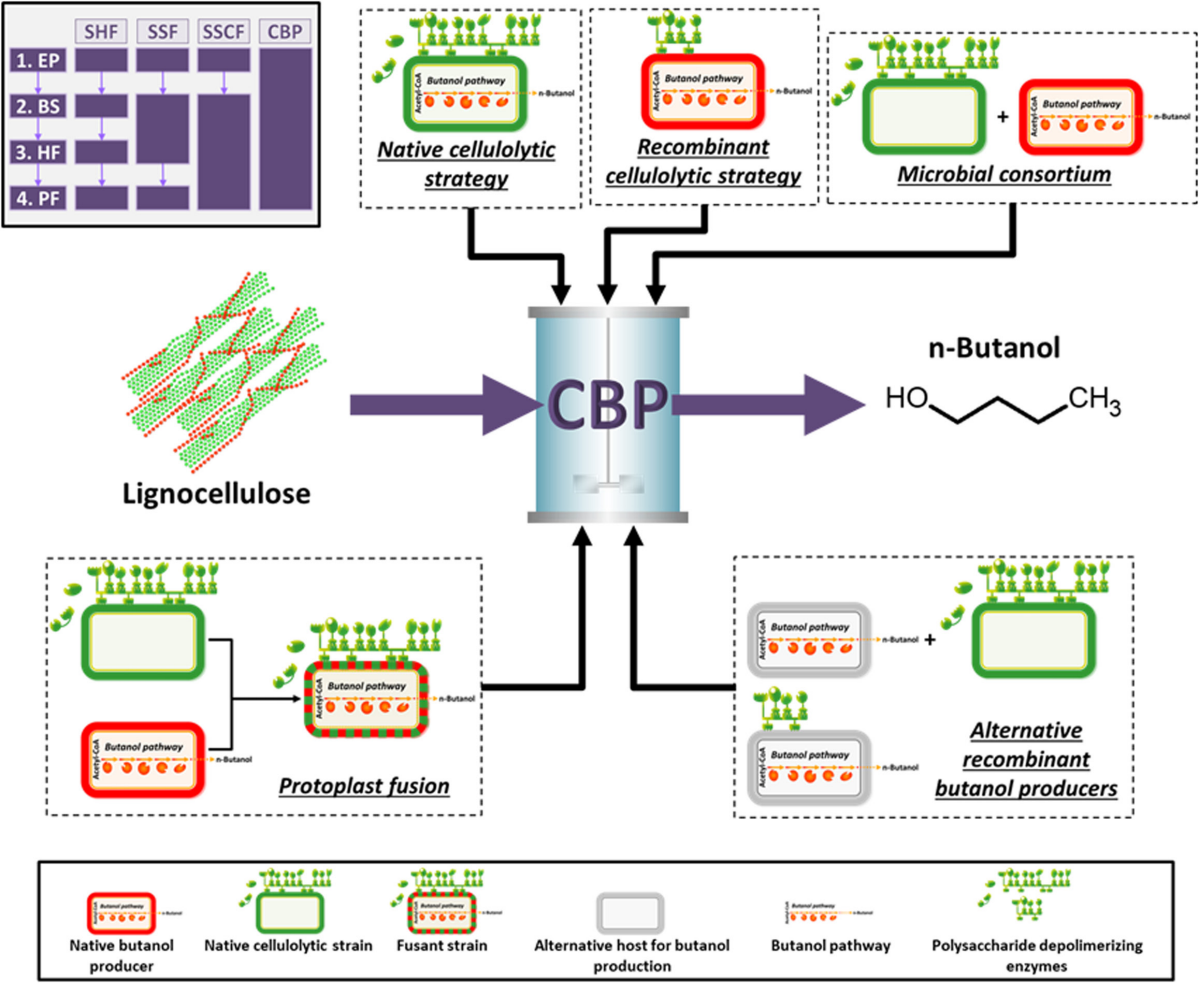
Strategies for consolidated bioprocessing (CBP) of lignocellulosic biomass to butanol. Lignocellulose biorefining includes four biological events, that is, cellulase and hemicellulase production (EP), biomass saccharification (BS), hexose fermentation (HF), and pentose fermentation (PF). Depending on the degree of consolidation of these steps, process configuration is schematized as separate hydrolysis and fermentation (SHF), simultaneous saccharification and fermentation (SSF), simultaneous saccharification and co‐fermentation (SSCF), or CBP (upper left box). Five different approaches towards the development of CBP have been reported so far, namely native cellulolytic strategies, recombinant cellulolytic strategies, artificial microbial consortia, fusant strains, and alternative recombinant butanol producers (see text for further details).

Lignocellulose is significantly more recalcitrant to biodegradation than traditional ABE fermentation feedstocks. The four main native butanol producers, *C. acetobutylicum*, *Clostridium beijerinckii*, *Clostridium saccharobutylicum*, and *Clostridium saccharoperbutylacetonicum* (Gu et al., 2011, 2014), cannot directly grow on lignocellulose (Lee et al., 1985; Levi Hevroni et al., 2020; Sabathé et al., 2002; Sankar et al., 2003). Therefore, inefficient multistep processes are required for fermenting lignocellulose to butanol which features biomass pre‐treatment and/or dedicated cellulase production and/or separated biomass saccharification and/or hexose and/or pentose fermentation (Figure 1) (Mazzoli, 2020; Tarraran & Mazzoli, 2018). Maximum butanol titer (14.5 g L^−1^) (Qureshi et al., 2010) and productivity (0.36 g L^−1^ h^−1^) (Gao et al., 2014) obtained by fermentation of lignocellulose hydrolysates is generally significantly lower than that achieved on starchy biomass or molasses (Abo et al., 2019; Birgen et al., 2019; Zhao et al., 2019). More importantly, process costs (especially those related to biomass pretreatment and exogenous cellulase supplementation) dramatically reduce the economic competitiveness of this approach (Jiang et al., 2015).

The present paper aims to provide an overview of the substantial research activity which has been dedicated to developing single‐step fermentation (namely consolidated bioprocessing, CBP) of lignocellulosic biomass to butanol. The potential reduction of capital and operating costs associated with CBP has been estimated between 40% and 77% with respect to alternative process configurations (i.e., simultaneous saccharification and fermentation, SSF, or simultaneous saccharification and co‐fermentation, SSCF) (Figure 1) (Lynd et al., 2005, 2008). To date, this aim has been pursued by five alternative approaches (Figure 1):

- The native cellulolytic strategy (NCS) aims at introducing and/or improving butanol production in natural (hemi)cellulolytic strains (e.g., *Clostridium cellulovorans* and *Clostridium thermocellum*) (Bao et al., 2021; Mazzoli & Olson, 2020).
- The recombinant cellulolytic strategy (RCS) focuses on equipping native butanol‐producing microorganisms (e.g., *C. acetobutylicum*) with the ability to directly ferment (hemi)cellulose (Soucaille et al., 2010; Willson et al., 2016).
- Artificial microbial consortia of (hemi)cellulolytic and solvent‐producing strains (Jiang et al., 2020; Wen et al., 2017).
- Development of strains through the fusion of protoplasts of (hemi)cellulolytic and solvent‐producing microorganisms (Begum & Dahman, 2015; Syed & Dahman, 2015).
- Engineering (hemi)cellulolytic and/or butanol‐producing phenotype in other suitable microbial paradigms (e.g., showing high genetic tractability or high butanol tolerance) (Shen et al., 2011; Zhang et al., 2018).

Metabolic engineering approaches related to NCS, RCS, and the development of other microbial strains combining direct fermentation of (hemi)cellulose and butanol production have generally been based on genetic manipulation. For each of these five strategies, the advances obtained so far will be summarized in a dedicated section.

In addition, the development of high‐performing butanol producers is challenged by butanol cell toxicity, which is higher than other established biofuels, such as ethanol (Ingram, 1976). This hampers solvent titers even in the traditional ABE fermentation thus increasing the capital and operational process cost (Vane, 2008). Although this issue is not specific to the production of cellulosic butanol, the last section of this review will summarize strategies and current advances in the development of butanol‐hypertolerant strains.

## DEVELOPMENT OF MICROBIAL STRAINS FOR PRODUCTION OF (HEMI)CELLULOSIC BUTANOL BY CBP

### NCS: improvement of butanol production in native cellulolytic microorganisms

There are few reports of cellulolytic microorganisms that can naturally produce butanol (Li et al., 2018; Mendez et al., 1991; Virunanon et al., 2008). The most well‐documented strain is *Thermoanaerobacterium thermosaccharolyticum* TG57 which can ferment microcrystalline cellulose or xylan and generate butanol as the main product although at low efficiency (titer ≤ 3.6 g L^−1^, yield ≤ 0.23 g g^−1^, and productivity ≤ 0.019 g L^−1^ h^−1^) (Li et al., 2018). Similar butanol production has been reported on xylan‐fermenting clostridia (Li & He, 2016; Xin et al., 2017). To the best of our knowledge, no attempt has been performed to improve butanol production in these strains by metabolic engineering.

So far, butanol production has been *de novo* introduced in three cellulolytic clostridia, namely the mesophilic *Clostridium cellulolyticum* (Gaida et al., 2016) and *C. cellulovorans* (Yang et al., 2015), and the thermophilic *C. thermocellum* (Tian, Conway, et al., 2019) as recently reviewed (Cheng et al., 2019; Mazzoli & Olson, 2020; Wen, Li, Liu, Jin, & Yang, 2020; Xin et al., 2019). Efficient plant biomass fermentation by these strains and/or established understanding of their metabolism and/or availability of genetic tools have likely promoted research on these paradigms (Bao, Zhao, Zhang, & Yang, 2019b; Mazzoli & Olson, 2020). Metabolic engineering strategies used on these microorganisms rely on the butanol pathway of solventogenic clostridia.

**FIGURE 2:**
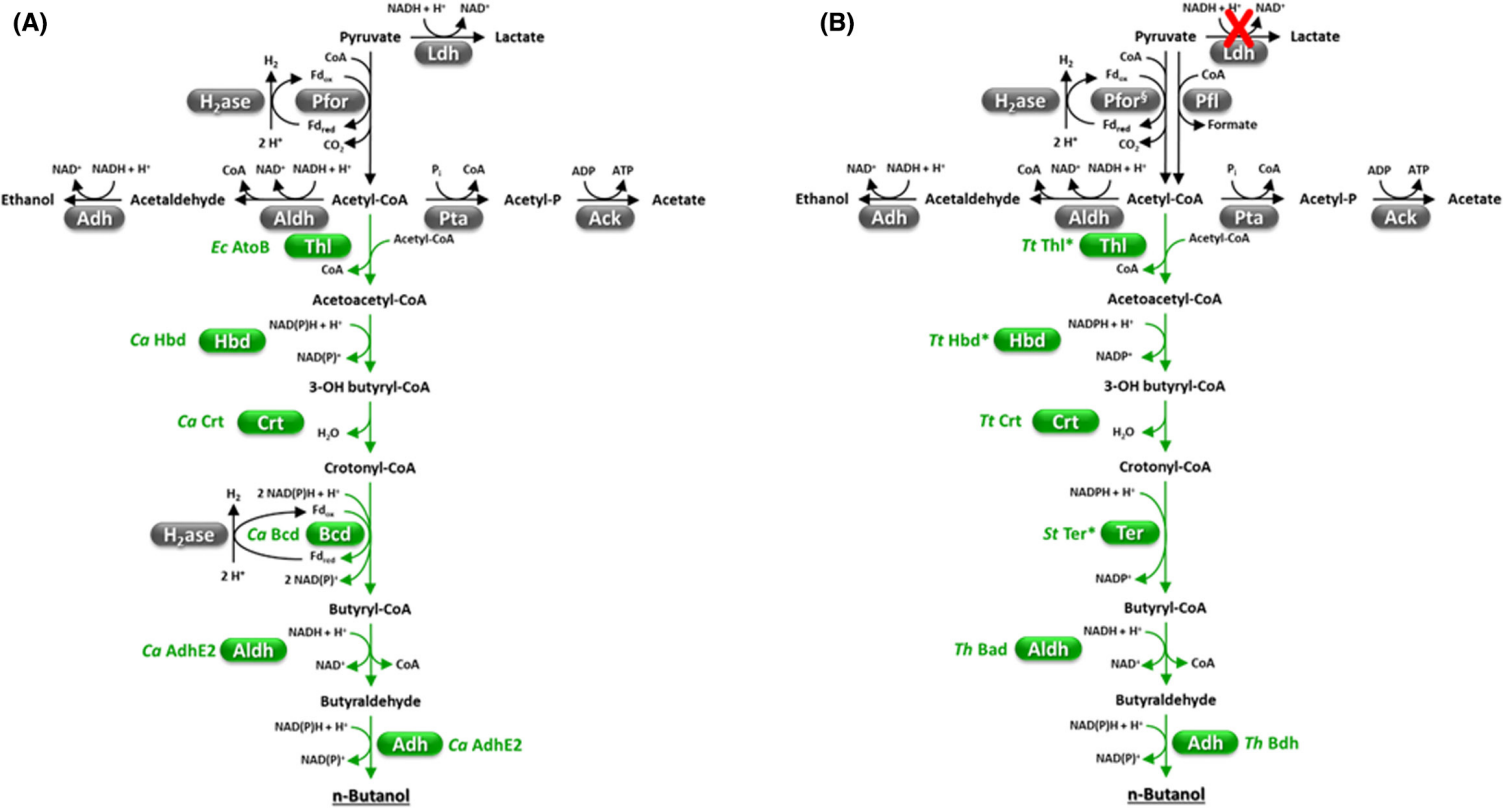
Butanol pathways engineered in *Clostridium cellulolyticum* (A) and *Clostridium thermocellum* (B) (Gaida et al., 2016; Tian, Conway, et al., 2019). Heterologous enzymes and the reactions they catalyze are indicated in green. As regards *C. thermocellum*, only the combination of gene/protein modifications that led to the highest butanol production is shown (Tian, Conway, et al., 2019). The latter also includes: (i) disruption of *ldh* (red cross) and *pfor4* (Pfor^§^) genes (involved in lactate and isobutanol production, respectively); (ii) optimization of Thl, Hbd, and Ter by protein engineering (indicated by an asterisk, see text for further details). Abbreviations: *St*, *Spirochaeta thermophila*; *Th*, *Thermoanaerobacter* sp. X514; *Tt*, *Thermoanaerobacter thermosaccharolyticum*.

The whole acetyl‐CoA‐to‐butanol pathway was introduced in *C. cellulolyticum* (Gaida et al., 2016) and *C. thermocellum* (Tian, Conway, et al., 2019) (Figure 2). Substantial genetic engineering efforts were performed especially on *C. thermocellum* which included: (i) assembly of twelve different thermophilic butanol pathway permutations; (ii) disruption of some parasitic pathways (i.e., lactate and isobutanol production); and (iii) optimization of some key enzymes, namely catalytic efficiency of thiolase (Thl) and cofactor preference (from NADH to NADPH) of 3‐hydroxybutyryl‐CoA dehydrogenase (Hbd) and trans‐enoyl‐CoA reductase (Ter) (Figure 2B) (Tian, Conway, et al., 2019). However, very low butanol titers (<0.5 g L^−1^) were obtained through fermentation of crystalline cellulose by engineered *C. cellulolyticum* or *C. thermocellum* (Gaida et al., 2016; Tian, Conway, et al., 2019). Inefficient or imbalanced expression of butanol pathway enzymes, low enzyme stability, limited co‐factor availability, and unfavorable reaction thermodynamics have likely contributed to a variable extent to low butanol production in these strains (Gaida et al., 2016; Tian, Conway, et al., 2019) as previously reported in other engineered butanol producers (Nielsen et al., 2009; Shen et al., 2011).

**FIGURE 3:**
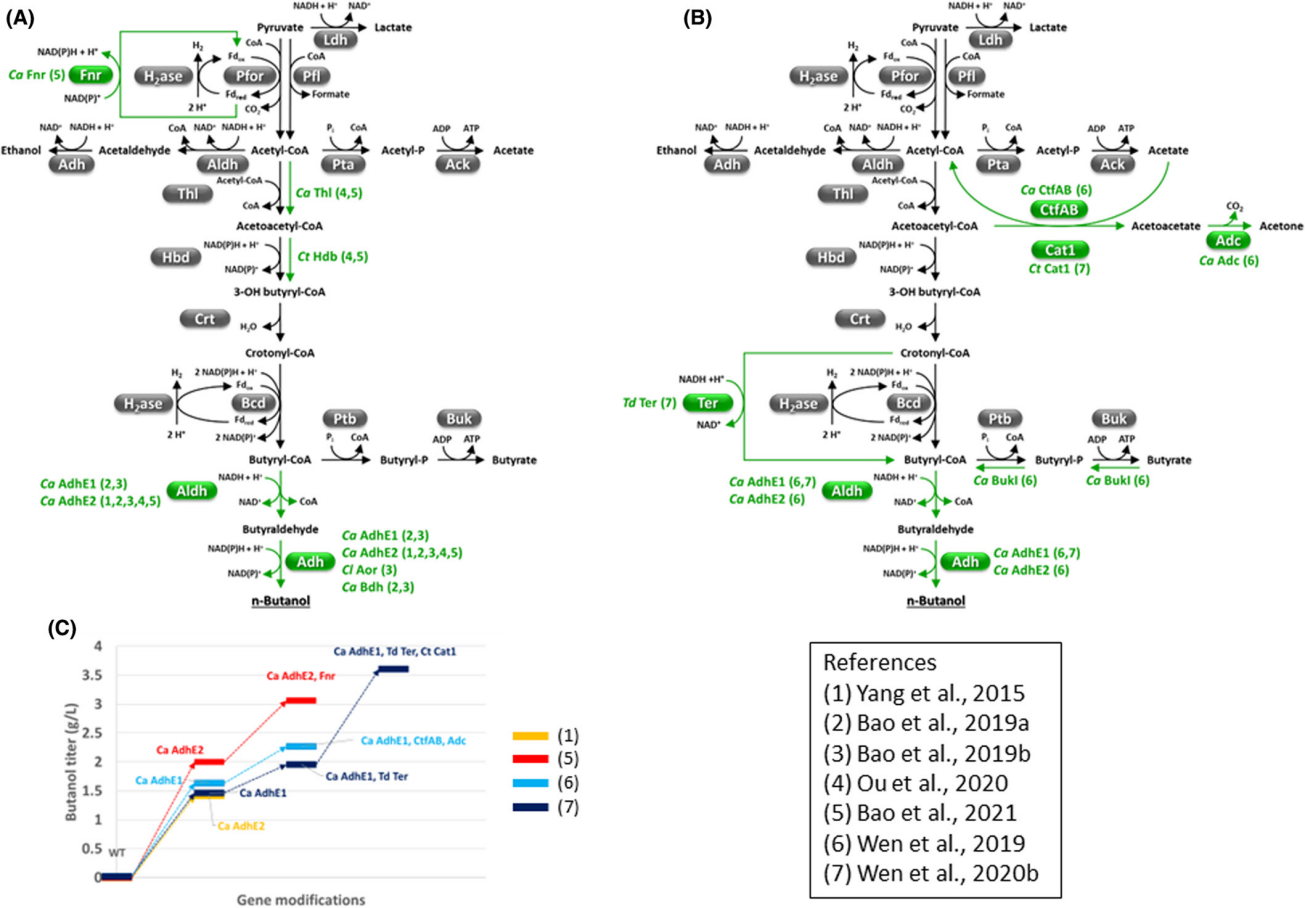
Butanol pathways engineered in *Clostridium cellulovorans*. Independent metabolic engineering strategies were developed at the Ohio State University (USA, A) and the Chinese Academy of Sciences (China, B). Heterologous enzymes and the reactions they catalyze are indicated in green. The numbers in brackets refer to the studies in which the gene modifications were reported. (B) Acid re‐assimilation by CftAB is coupled to the production of acetoacetate (hence, requires co‐expression of Adc) while the reaction catalyzed by Cat1 is not. (C) The gene modifications that were more effective in increasing butanol titer in *C. cellulovorans* are reported. Abbreviations: Aor, aldehyde ferredoxin oxidoreductase; *Cl*, *Clostridium ljungdahlii*.

Higher butanol titers were reported through fermentation of cellulose (i.e., 3.06 g L^−1^) (Bao et al., 2021) or alkali‐extracted corn cobs (i.e., 4.96 g L^−1^) (Wen, Ledesma‐Amaro, Lu, Jin, & Yang, 2020) by engineered *C. cellulovorans*. More limited genetic modification was required for engineering butanol production in this bacterium since it is naturally equipped with a butyryl‐CoA (a butanol precursor) pathway (Figure 3) (Wen et al., 2019; Yang et al., 2015). Metabolic engineering strategies developed at the Ohio State University (USA) and the Chinese Academy of Sciences (China) were based on: (i) overexpression of heterologous bifunctional alcohol‐aldehyde dehydrogenase (e.g., *C. acetobutylicum* AdhE1 and AdhE2) to convert butyryl‐CoA to butanol (Bao, Zhao, Li, et al., 2019; Wen et al., 2019) and (ii) enhancement of acetyl‐CoA to butyryl‐CoA flux to improve the C_4_/C_2_ fermentation product ratio (Figure 3). As regards the latter strategy, overexpression of either Ter from *Treponema denticola* (Wen, Ledesma‐Amaro, Lu, Jin, & Yang, 2020) or heterologous Thl and Hbd (Bao et al., 2021; Ou et al., 2019) was effective in decreasing C_2_ product (that is ethanol and/or acetate) yield. However, Ter expression more selectively enhanced butanol (and, to a similar extent, butyrate) production (≈26%) (Wen, Ledesma‐Amaro, Lu, Jin, & Yang, 2020), while overexpression of Thl and/or Hbd mainly led to butyrate accumulation (Bao et al., 2021; Ou et al., 2019). Ter catalyzes NADH‐dependent reduction of crotonyl‐CoA to butyryl‐CoA which is thermodynamically more favorable (Δ_r_G'°= −50.6 KJ/mol, at pH = 7.5) and less NADH‐consuming than the reaction catalyzed by butyryl‐CoA dehydrogenase (Bcd) which requires 2 NADH and oxidized Ferredoxin (Fd) (Δ_r_G'°= −37.3 KJ/mol, at pH = 7.5) (Flamholz et al., 2012; Wen, Ledesma‐Amaro, Lu, Jin, & Yang, 2020). However, it is worth remembering that attempts to disrupt *C. cellulovorans* genes encoding Bcd complex and functionally replace this enzyme with Ter were so far unsuccessful (Wen, Ledesma‐Amaro, Lu, Jin, & Yang, 2020).

Additional metabolic engineering strategies employed to improve butanol production in *C. cellulovorans* include:

- *Re‐assimilation of acetic and butyric acid*, thus mimicking the metabolism of more established solventogenic clostridia. Introduction of acetone production‐uncoupled pathway (i.e., *Clostridium tyrobutyricum* butyryl‐CoA−acetate CoA transferase, Cat1) (Wen, Ledesma‐Amaro, Lu, Jin, & Yang, 2020) led to higher improvement of butanol titer (≈70% vs. 38%) with respect to acetone production‐coupled reactions (namely *C. acetobutylicum* CoA transferase, CtfAB, and acetoacetate decarboxylase, Adc) (Figure 3B) (Wen et al., 2019) and avoided acetone accumulation.
- *Expression of a ferredoxin NAD(P)* ^+^ *oxidoreductase* (Fnr, Fd_red_ + NAD(P)^+^ ⇆ Fd_ox_ + NAD(P)H + H^+^). Expression of *C. acetobutylicum* Fnr in addition to AdhE2 enhanced butanol titer by ≈50% (butanol titer = 3.06 g L^−1^) (Bao et al., 2021). Supplementation of methyl viologen (an artificial electron donor) to cultures of *C. cellulovorans adhE2‐fnr* on crystalline cellulose further increased butanol production up to 5.74 g L^−1^ (Bao et al., 2021), which is the highest cellulosic butanol titer reported so far for a process employing a single microorganism. These observations, together with the beneficial effect of introducing Ter reaction, suggest that the availability of reducing equivalents, with special attention to NADH, is a key factor for increasing butanol production in *C. cellulovorans* (Bao et al., 2021; Yang et al., 2015).
- *Improvement of pentose utilization*. This strategy increased *C. cellulovorans* growth on complex lignocellulosic biomass (alkali‐extracted corn cobs) and led to 37% higher butanol accumulation (final titer = 4.96 g L^−1^) (Wen, Ledesma‐Amaro, Lu, Jin, & Yang, 2020).

Even maximum butanol titers (5.74 g L^−1^) obtained by engineered *C. cellulovorans* are still far lower than those obtained by traditional ABE fermentation or by fermentation of lignocellulosic hydrolysates by native butanol producers (Abo et al., 2019; Birgen et al., 2019; Zhao et al., 2019). Further improvement of butanol production in cellulolytic clostridia seems possible also taking into account the recent improvement of *C. thermocellum* and *C. cellulovorans* tolerance to this solvent up to 12–15 g L^−1^ (Tian, Cervenka, et al., 2019; Wen et al., 2019):

- *Improvement of butyryl‐CoA pathway*. Thl, Bcd, and Hbd reactions are among the most challenging of the clostridial butanol pathway (Figures 2 and 3) because of unfavorable thermodynamics (Bcd, Thl) (Flamholz et al., 2012) and/or high NADH consumption (Bcd, Hbd) and/or issues related to heterologous enzyme expression (Bcd) (Shen et al., 2011; Tian, Conway, et al., 2019). A more systematic replacement of (a) Thl with acetyl‐CoA acetyltransferases having higher catalytic efficiency and/or lower sensitivity to CoA inhibition (e.g., *Escherichia coli* AtoB) (Nguyen et al., 2018); (b) Bcd with native or engineered Ter enzymes (Shen et al., 2011; Tian, Conway, et al., 2019); (c) NADH‐dependent Hbd and/or Ter with NADPH‐dependent counterparts (Nguyen et al., 2018; Tian, Conway, et al., 2019) seems recommended.
- *Disruption of pathways that compete for carbon intermediates and/or reducing equivalents*. Reliable gene manipulation tools are available for disrupting acetate, formate, ethanol, and H_2_ production in *C. thermocellum* (Mazzoli & Olson, 2020). Currently, the efficiency of genetic tools developed for *C. cellulovorans* and *C. cellulolyticum* is more limited (Li et al., 2012; Wen et al., 2017; Xu, Li, et al., 2017). It is worth remembering that attempts to eliminate butyrate production in *C. cellulovorans* were so far unsuccessful (Wen et al., 2019; Wen, Ledesma‐Amaro, Lu, Jin, & Yang, 2020).
- *Enhancement of NADH production routes*. Implementing enhanced electron transfer from Fd to pyridine cofactors, for example, by overexpressing Fnr enzymes (Buckel & Thauer, 2013), has been used to improve the production of ethanol in *C. thermocellum* (Lo et al., 2017) and butanol in *C. cellulovorans* (Bao et al., 2021). This strategy could be more extensively used to increase butanol production in cellulolytic microorganisms. The expression of heterologous formate dehydrogenase (Fdh, formate + NAD^+^ → CO_2_ + NADH + H^+^) could provide an additional source of NADH (Shen et al., 2011).
- *Dysregulation of cellular ATP level*. Reduction of intracellular ATP levels (e.g., by overexpressing ATP hydrolyzing components of F_1_F_0_‐ATPase) promoted 14.5% higher solvent titer by *C. acetobutylicum* (Dai et al., 2021) and also enhanced the glycolytic flux (through relief of allosteric inhibition of some glycolytic enzymes) in other microorganisms (Dai et al., 2021).
- *Dysregulation of cellular redox homeostasis*. The butyryl‐CoA pathway of clostridia is generally downregulated under a low intracellular NADH/NAD^+^ ratio through the transcriptional repressor Rex (Hu et al., 2016; Nguyen et al., 2018). As this seems to occur also in *C. cellulovorans* (Costa et al., 2021), it would be worth testing if disruption of the *rex* gene may increase butanol production in this strain as previously reported in *C. acetobutylicum* (Nguyen et al., 2018).

Apart from these cellulolytic clostridia paradigms, the recent development of efficient gene manipulation tools for the thermophilic cellulolytic fungus *Myceliophthora thermophila* makes this microorganism a further promising candidate for heterologous butanol production (Gu et al., 2018; Li, Lin, et al., 2020).

### RCS: engineering plant polysaccharide depolymerizing activity in native butanol producers

Studies aimed at engineering cellulolytic phenotype in non‐native hosts have generally been based on mimicking two main natural cellulase system paradigms, namely the non‐complexed enzyme model of aerobic fungi and bacteria, or the cellulosome complexes of anaerobic microbes (Lynd et al., 2002). Cellulosomes generally benefit from higher synergistic activity due to closer proximity between the enzymatic subunits and between these and the microbial cell (Artzi et al., 2017). These complexes generally include scaffolding proteins (i.e., scaffoldins) that can bind enzyme subunits (through strong interaction between scaffoldin‐beared cohesin and enzyme‐beared dockerin domains), cellulose (through carbohydrate‐binding modules, CBM), and microbial cell surface (through specific domains that mediate covalent or non‐covalent linkages) (Artzi et al., 2017; Mazzoli et al., 2012). Within this research area, most studies focused on engineering native butanol‐producing microorganisms have been carried out on *C. acetobutylicum* ATCC 824. As far as we know, only two studies reported expression of cellulases in other ABE fermenting strains which improved *C. beijerinckii* direct fermentation of lichenan to solvents (including butanol) but did not enable this strain to grow on carboxymethyl cellulose (CMC) or crystalline cellulose (López‐Contreras et al., 2001; Quixley & Reid, 2000).

Although *C. acetobutylicum* ATCC 824 cannot grow on CMC or Avicel (crystalline cellulose), its genome encodes several plant‐polysaccharide depolymerizing enzymes including a cellulosome gene cluster (Nölling et al., 2001) which provide this microorganism with some extracellular hydrolytic activity on amorphous cellulose (i.e., CMC and phosphoric acid swollen cellulose, PASC) (López‐Contreras et al., 2003, 2004; Sabathé et al., 2002). The observation that a closely related strain (i.e., *C. acetobutylicum* NRRL B 527) can hydrolyze Avicel and acid‐swollen cellulose has suggested that *C. acetobutylicum* ATCC 824 could have lost its cellulolytic phenotype over many years of growth under laboratory conditions without selective pressure for cellulose utilization (Sabathé et al., 2002). Since 2004, substantial progress in the “repair” of the defective cellulase system of *C. acetobutylicum* ATCC 824 has been obtained through the expression of cellulosomal components derived from phylogenetically related cellulolytic clostridia. These studies showed that the expression of heterologous cellulosomal enzymes in *C. acetobutylicum* is highly challenging (Kovács et al., 2013; Mingardon et al., 2005, 2011; Willson et al., 2016) consistent with similar observations reported on other microbial hosts (Tarraran et al., 2021; Xu et al., 2018). Certain glycoside hydrolases (GHs)/GH classes (e.g., *C. cellulolyticum* Cel9E, Cel9G, and Cel48F, that is those with larger catalytic modules or additional domains) showed higher toxicity (Mingardon et al., 2011; Willson et al., 2016). However, secretion of truncated forms (i.e., lacking the dockerin domain) has frequently been reported also for other cellulosomal enzymes (Mingardon et al., 2005, 2011). In addition, attempts to introduce larger scaffoldins, that is containing a higher number of cohesins (and therefore able to bind a greater number of enzymes and generate higher efficient complexes), resulting in lower protein expression yields (Cha et al., 2007; Kovács et al., 2013; Krauss et al., 2012).

Despite these hurdles, the expression of a cell‐wall anchored trifunctional minicellulosome in *C. acetobutylicum* was achieved in 2016 (Willson et al., 2016). Strains expressing some of the main *C. cellulolyticum* cellulosomal enzymes (the processive endocellulase Cel48F, the endoglucanase Cel9G and the xylanase Xin10A) showed improved hydrolysis of PASC and wheat straw but were not able to grow on these substrates. The main phenotypic improvement (which was essentially linked to XynA10 expression) was the enhancement of direct xylan fermentation which led to the production of 1.36 g L^−1^ butanol (Willson et al., 2016). A patent filed in 2010 seemed to have achieved more significant progress through a more minimalist approach (Soucaille et al., 2010). As *C. acetobutylicum* native Cel9C and Cel9X cellulases show specific activity similar to their *C. cellulolyticum* homologous, the study focused only on replacing the inactive catalytic module of endogenous Cel48A with the homologous domain from *C. cellulolyticum* Cel48F. After further strain improvement by adaptive evolution, the engineered *C. acetobutylicum* could efficiently ferment PASC to a mixture of butanol, acetone, ethanol, acetic, and butyric acid (Soucaille et al., 2010). Unfortunately, no further detail about butanol production (titer, yield, and productivity) by this strain on PASC is currently available. Nor it is known if this strain can grow on more recalcitrant cellulosic substrates (e.g., Avicel).

As far as we know, no further progress has been reported after 2016 towards engineering a *C. acetobutylicum* able to directly ferment crystalline cellulose and real lignocellulosic feedstocks. Challenges include the extreme sophistication of the native cellulase systems (Bule et al., 2018; Galera‐Prat et al., 2020; Leis et al., 2017; Xu, Huang, et al., 2015) (together with the high complexity of lignocellulosic substrates) which makes it difficult to mimic their efficiency through designer cellulosomes. Furthermore, insufficient understanding of the mechanisms promoting cellulase secretion (De Paula et al., 2019; Yan & Wu, 2013, 2014), as well as the complexity and species‐specificities of protein secretion mechanisms are still major barriers towards rational engineering of recombinant cellulolytic strains. The findings by Soucaille et al. (2010) suggest that future strategies should focus more on improving the native *C. acetobutylicum* cellulosome by targeted complementation of missing or deficient activities. An increase in the enzyme‐display level and direct fermentation of crystalline cellulose has sometimes been achieved in other hosts by expressing multiple scaffoldins (cell surface‐anchoring and adaptor), thus avoiding the issue of secreting large scaffoldins (Anandharaj et al., 2020; Fan et al., 2012). Artificial syntrophic consortia (consisting of recombinant strains that secrete single/few different cellulosomal subunits) have allowed decreasing the burden on the cellular machinery of each strain and maximize heterologous protein expression (Anandharaj et al., 2020; Stern et al., 2018). These approaches could help further advances in engineering cellulolytic *C. acetobutylicum*.

### Engineering artificial consortia composed of (hemi)cellulolytic and solventogenic microorganisms

The development of artificial consortia consisting of (hemi)cellulolytic microorganisms and solvent‐producing strain(s) is an interesting alternative for developing CBP of lignocellulosic biomass to butanol that avoids or reduces the need for complicated genetic and metabolic engineering. In natural environments, 99% of microorganisms exist as microbial consortia (Ding et al., 2016) which can perform more complicated tasks than single microbial strains by compartmentalizing functions in different strains (Cui et al., 2021; Ding et al., 2016; Xin et al., 2019). However, designing and maintaining stable artificial microbial communities leading to high product formation is frequently challenging (Cui et al., 2021; Jiang et al., 2019; Johns et al., 2016) owing to possibly different conditions (e.g., temperature, pH, and pO_2_) for microbial growth or metabolic activity (e.g., cellulase catalysis vs. butanol production) or different growth kinetics of the microbial partners. These aspects are particularly challenging at the industrial scale (Cui et al., 2021; Jiang et al., 2019; Johns et al., 2016). Even at the laboratory scale, inconsistencies in these features may be difficult to fix or have been reduced by also integrating metabolic engineering approaches (Wen et al., 2017; Wen, Ledesma‐Amaro, Lu, Jiang, et al., 2020).

So far, several studies have reported the development of artificial microbial consortia aimed at CBP of lignocellulosic feedstocks to butanol (Table 1). In most cases, bacteria belonging to the *Clostridium* genus have been employed, although other hemicellulolytic bacteria (e.g., *Kluyvera sp*. OM3, *Thermoanaerobacterium thermosaccharolyticum*) (Jiang et al., 2018, 2020; Xin & He, 2013) or white rot fungi (*Phlebia sp*. MG‐60‐P2) (Tri & Kamei, 2020) have sometimes been used as lignocellulose depolymerizing strains (Table 1). However, most studies refer to the thermophilic *C. thermocellum* or the mesophilic *C. cellulovorans* as the cellulolytic member of the consortium (Table 1). Indeed, *C. thermocellum* shows one of the highest efficiencies of cellulose solubilization (Argyros et al., 2011; Demain et al., 2005; Lynd et al., 2002), while *C. cellulovorans* can metabolize a large panel of plant polysaccharides such as cellulose, hemicelluloses, and pectins (Aburaya et al., 2015, 2019). Apart from the microorganisms of choice, studies differ as regards the fermentation mode (batch and fed‐batch), feedstock, temperature, use of pH regulation, time of inoculum of the solventogenic strain, and use of butyrate supplementation (for triggering butanol production), which is partly related to the microorganisms used (e.g., growth temperature) (Table 1). In most cases, the inoculum of the butanol‐producing microorganism was delayed so as to allow sufficient biomass depolymerization and accumulation of soluble carbohydrates by the (hemi)cellulolytic strain (Table 1). This choice was forced when partners with different growth temperatures were used, such as in studies employing *C. thermocellum* (Jiang et al., 2018, 2020; Kiyoshi et al., 2015; Nakayama et al., 2011; Wen et al., 2014a; Yu et al., 1985). Furthermore, anaerobic cellulolytic bacteria generally prefer pH close to 7 and cannot grow at pH < 6 (Usai et al., 2020; Whitham et al., 2018; Wu et al., 2017), while the production of solvents in clostridia is generally triggered by acidic pH (Dai et al., 2021; Yang et al., 2013). Therefore, biphasic modes of fermentation featuring different temperatures and/or pH enabling optimal growth/activity of the microbial partners have frequently been used resulting in longer fermentation time and lower productivities.

**TABLE 1:** Butanol production through direct fermentation of lignocellulosic biomass by artificial microbial consortia

Strains	Substrate	Fermentation mode	Time delay before solventogenic strain inoculum (h)	pH regulation	Temperature	Titer (g L^−1^)	Yield (g g^−1^)	Productivity (g L^−1^ h^−1^)	References
*Clostridium cellulolyticum* + *Clostridium acetobutylicum* NCIB 619	Solka floc cellulose	Fed‐batch	48	7.0 (48h), then 6.0	35°C	0.8	0.03	0.003	Petitdemange et al. (1983)
*Clostridium thermocellum + C. acetobutylicum*	Solka floc cellulose + butyrate	Batch	72	No	60°C (72 h), then 37°C	2.4	0.18^a^	0.014	Yu et al. (1985)
*Kluyvera sp*. OM3 + *Clostridium sp*. BOH3	Birchwood xylan	Batch	72	No	35°C	1.2	≈0.03^b^	0.008	Xin and He (2013)
*C. thermocellum* ATCC 27405 + *Clostridium saccharoperbutylacetonicum* ATCC 13564	Avicel cellulose	Batch	≥ 24	No	60°C (≥24 h), then 30°C	7.9	≈0.20^c^	0.030	Nakayama et al. (2011)
*C. thermocellum* ATCC 27405 + *Clostridium beijerinckii* NCIMB 8052	Alkali extracted corn cob	Fed‐batch	96	7.5 (60 h), then pH 6.0 (96 h), then no regulation	60°C (96 h) then 33°C	10.9	0.12	0.056	Wen et al. (2014a)
*Clostridium celevecrescens* N3‐2 + *C. acetobutylicum* ATCC 824	Filter paper cellulose	Batch	48	No	37°C	2.7	0.13	0.014	Wang et al. (2015)
*Clostridium cellulovorans* 743B + *C. beijerinckii* NCIMB 8052	Alkali extracted corn cob	Fed‐batch	No	7.0 (24 h), then no regulation	37°C	8.3	0.12	0.104	Wen et al. (2014b)
*C. thermocellum NBRC 103400* + *C. saccharoperbutylacetonicum strain N1‐4*	Delignified rice straw	Batch	24	No	60°C (24 h), then 30°C	5.5	0.03	0.138	Kiyoshi et al. (2015)
^d^ *C. cellulovorans* 743B [∆(*ldh*, *ack)* i‐*hydA* +(*Cc buk*)] + *C. beijerinckii* NCIMB 8052 [∆*xylR* +(*Cb xylT Cb ctfAB*)]	Alkali extracted corn cob	Fed‐batch	No	7.0 (34.5 h), then no regulation	37°C	11.5	0.14	0.106	Wen et al. (2017)
*Thermoanaerobacterium sp*. M5 + *C. acetobutylicum* NJ4	Xylan	Batch	72	7.5 (72 h), then 6.0	55°C (72 h), then 37°C	8.3	0.14	0.050	Jiang et al. (2018)
*C. cellulovorans* 743B + *C. beijerinckii* NCIMB 8052	Orange strained lees	Batch	384	No	37°C	n.a.	0.05	n.a.	Tomita et al. (2019)
*Thermoanaerobacterium thermosaccharolyticum M5* + *C. acetobutylicum NJ4*	Xylan	Batch	50	6.5 (50 h), then 5.5	55 (50 h) then 37	13.3	0.26	0.079	Jiang et al. (2020)
*T. thermosaccharolyticum M5* + *C. acetobutylicum NJ4*	Untreated corn cob	Batch	48	6.5 (48 h), then 5.5	55°C (48 h), then 37°C	7.6	0.12	0.045	Jiang et al. (2020)
^e^evolved *C. cellulovorans* 743B ∆*spo0A* ∆(Clocel_0798, Clocel_2169) +(*Ca adhE1*, *Cb augA*)+ *C. beijerinckii* NCIMB 8052	Alkali extracted corn cob	Batch	No	No	37°C	3.9	0.13	0.047	Wen, Ledesma‐Amaro, Lu, Jiang, et al. (2020)
*Phlebia sp*. MG‐60‐P2 (∆*pdc*) + *C. saccharoperbutylacetonicum*	Unbleached hardwood kraft pulp	Batch	120	No	28°C (120 h) then 30°C	3.2	n.a.	0.012	Tri and Kamei (2020)

*Note*: For engineered strains, the symbols “∆” or “+” precede the name of the genes that were disrupted or overexpressed, respectively. The acronym next to the gene name refers to the microbial source of that gene. The symbol “≈” was used for approximate values that were calculated from data in the corresponding studies.

Abbreviation: n.a., not available.

^a^ Butanol yield was calculated with respect to consumed reducing sugars.

^b^ Butanol yield was calculated with respect to the initial concentration of xylan (40 g L^−1^).

^c^ Butanol yield was calculated with respect to the initial concentration of cellulose (40 g L^−1^).

^d^ In the engineered *C. cellulovorans* strain the hydrogenase encoding gene (*hydA*) was down‐regulated by CRISPR interference.

^e^ This consortium included the solvent‐producing *C. beijerinckii* NCIMB 8052 and *C. cellulovorans* 743B strain lacking the gene (*spo0A*), subjected to adaptive evolution for improved tolerance to acidic pH and further engineered by deletion of two cell wall lyases (Clocel_0798, Clocel_2169) and overexpression of the gene encoding *C. acetobutylicum* bifunctional alcohol‐aldehyde dehydrogenase AdhE1 and *C. beijerinckii* NCIMB 8052 agmatine deiminase (*Cb augA*).

The *C. thermocellum*–*C. beijerincki* consortium developed by Wen et al. (2014a) and the one consisting of *Thermoanaerobacterium* sp. M5/*C. acetobutylicum* conceived by Jiang et al. (2020) led to the highest butanol titers (i.e., ≈11–13 g/L) obtained so far through artificial consortia of natural microorganisms (Table 1). These titers are close to those obtained through fermentation of lignocellulose hydrolysates by solventogenic clostridia (Gu et al., 2011). However, the efficiency of ABE fermentation on starch or soluble sugars (i.e., ≈20 g L^−1^ titer, ≈0.33 g g^−1^ yield, and ≈0.5 g L^−1^ h^−1^ productivity) is still significantly higher (Abo et al., 2019; Gu et al., 2011; Wen et al., 2014a). The performance of microbial consortia strongly depends on the mutual benefit between strains (Song et al., 2014), which rely on the metabolic characteristics of the partners and the culture conditions. Based on the complexity of these biological systems, implementation of empirical strategies with model‐driven analysis seems desirable to rationally design more efficient and robust microbial consortia (Salimi et al., 2010; Yoo et al., 2015; Zomorrodi & Segrè, 2016).

More recently, similar butanol titer has been obtained also by a consortium consisting of engineered: (i) butyrate‐overproducing *C. cellulovorans* and (ii) *C. beijerinckii* showing increased re‐assimilation of organic acids and metabolism of pentoses (Wen et al., 2017). This consortium shows one of the highest butanol productivities (0.106 g L^−1^ h^−1^) through direct fermentation of lignocellulose (Table 1). This study shows that engineered microorganisms can be used to improve the degree of synergism of microbial consortia. Another interesting example was based on developing an acid‐resistant *C. cellulovorans* (it could tolerate pH 5.5) so as to allow a larger pH range compatible for simultaneous cellulose depolymerization by *C. cellulovorans* and sugar fermentation to butanol by *C. beijerinckii* (Wen, Ledesma‐Amaro, Lu, Jiang, et al., 2020) (Table 1). Direct fermentation of alkali extracted corn cobs to butanol by the *C. cellulovorans*–*C. beijerinckii* consortium was possible without the need for pH regulation. However, butanol titer was still lower than that obtained by other *C. cellulovorans*–*C. beijerinckii* consortia through two‐stage regulated pH fermentation (Wen, Ledesma‐Amaro, Lu, Jiang, et al., 2020).

### Development of strains for direct production of cellulosic butanol by protoplast fusion

Cell protoplast fusion may allow the improvement of microbial phenotype (e.g., product formation or stress tolerance) without the need for complicated genetic engineering (Chen et al., 2020; Hospet et al., 2021). This is particularly advantageous in the case of complex phenotypic traits and generates fusant strains whose application is not subject to limitations that regard genetically modified organisms (Chen et al., 2020). Recently, fusants derived from solventogenic *C. acetobutylicum* ATCC 4259 or *C. beijerinckii* ATCC BA101 and cellulolytic *C. thermocellum* ATCC 27405 have been obtained and tested for their ability to directly ferment dilute sulphuric acid‐pretreated wheat straw (Begum & Dahman, 2015; Syed & Dahman, 2015). This pretreatment is expected to partially hydrolyze the lignocellulosic biomass, especially the hemicellulosic component but leaves unhydrolyzed a significant part of polysaccharides (Begum & Dahman, 2015). Butanol titers generated by fusant fermentation of pretreated wheat straw were about 2‐fold higher than those produced by either culture of pure *C. acetobutylicum* or *C. beijerinckii* supplemented with commercial cellulase/hemicellulose mixture or co‐cultures of *C. thermocellum* and *C. acetobutylicum* or *C. beijerinckii* (Begum & Dahman, 2015; Syed & Dahman, 2015). Higher fermentation temperature (from 35°C to 45°C) resulted in further improvement of butanol production by fusants. The highest performing strain was the *C. beijerinckii*–*C. thermocellum* fusant which generated 14.13 g L^−1^ butanol at 0.29 g g^−1^ yield and 0.12 g L^−1^ h^−1^ productivity (Begum & Dahman, 2015). These results indicate the development of fusant strains as a further promising solution towards the production of cellulosic butanol by CBP. However, it is necessary to assess the reproducibility of these results and their exploitation potential. The success of protoplast fusion strategies is frequently threatened by the low efficiency of fusion, lack of high throughput screening methods for rapid identification of the desired phenotype, and low genetic stability of fusants (Magocha et al., 2018; Steensels et al., 2014).

### Other microbial candidates for cellulosic butanol production

Recombinant production of butanol has been explored in several non‐native hosts such as *E. coli* (Ferreira et al., 2020), *Bacillus subtilis* (Nielsen et al., 2009), *C. tyrobutyricum* (Bao et al., 2020), lactic acid bacteria (Li et al., 2021), *Pseudomonas putida* (Nielsen et al., 2009), and *Saccharomyces cerevisiae* (Azambuja & Goldbeck, 2020). Interest in using these alternative microbial platforms is motivated by their higher genetic tractability (e.g., *E. coli* and *S. cerevisiae*) and/or tolerance to butanol (e.g., lactic acid bacteria, *P. putida*, and *Bacillus* sp.) and/or robustness under industrial conditions (e.g., *E. coli* and *S. cerevisiae*). To date, butanol titers obtained by metabolic engineering of these microorganisms were generally lower than 1 g L^−1^ (Table 2). However, studies on *E. coli* (Shen et al., 2011), *C. tyrobutyricum* (Zhang et al., 2018), and the unconventional yeast *Arxula adeninivorans* (Kunze & Haehnel, 2011) resulted in butanol production efficiency similar to or higher than that of native producers (that is butanol titer ≥20 g L^−1^) (Table 2). More in detail, an engineered *E. coli* strain was able to produce about 30 g L^−1^ butanol in fed‐batch fermentation with continuous butanol removal (Shen et al., 2011). Strain engineering encompassed the introduction of a chimeric butanol pathway and increase of cellular NADH and acetyl‐CoA pools by disruption of genes encoding the native Adh, fumarate reductase, Ldh and Pta, and overexpression of a heterologous Fdh (Shen et al., 2011) (Table 2). The whole *C. acetobutylicum* acetyl‐CoA to butanol pathway was engineered in *A. adeninivorans* (Kunze & Haehnel, 2011). In addition, gene modification in this strain included overexpression of autologous *bad* and *bdh* genes, elimination of peroxisomal oxidation of butyryl‐units and glycerol production (Table 2). The engineered *A. adeninivorans* was reported to produce 20 g L^−1^ through fed‐batch fermentation of starch (Kunze & Haehnel, 2011). Much more limited gene modifications, namely inactivation of Cat1 and overexpression of *C. acetobutylicum* AdhE2, were necessary to convert the strong butyrate producer *C. tyrobutyricum* into a microorganism that mainly produces butanol (26.2 g L^−1^) (Zhang et al., 2018). An advantage of using recombinant butanol producers is that in these strains butanol production is uncoupled from the generation of other solvents (e.g., acetone and ethanol) such as in native ABE fermenters, which can potentially lead to higher butanol yield.

**TABLE 2:** Maximum production of butanol engineered in different non‐native “unconventional” producers

Strains	Gene modifications	Substrate	Fermentation mode	Titer (g L^−1^)	Yield (g g^−1^)	Productivity (g L^−1^ h^−1^)	References
*Arxula adeninivorans*	Δ(*cop*, *cpp*, *ctp*, *gpd1*) +*(Ca thl*, *Ca hbd*, *Ca crt*, *Ca bcd*, *Ca adhE2*, *Aa adhE2*, *Aa bdh*)	Starch	Fed‐batch	20	n.a.	n.a.	Kunze and Haehnel (2011)
*Bacillus subtilis*	Δ(*amyE*, *pyrD*, *thrC*) +*(Ca thl*, *Ca hbd*, *Ca crt*, *Ca bcd*, *Ca etfAB*, *Ca adhE2*)	Glycerol	Batch	0.024	0.005	<0.001	Nielsen et al. (2009)
*Clostridium tyrobutyricum*	Δ*cat1*, + *Ca adhE2*	Glucose	Batch	26.2	0.23	0.16	Zhang et al. (2018)
*Escherichia coli*	Δ(*adhE*, *frdBC*, *ldhA*, *pta)* +(*Cb fdh*, *Ec atoB*, *Ca hbd*, *Ca crt*, *Ca adhE2*, *Td Ter*)	Glucose	Fed‐batch + gas stripping	30	0.29	0.18	Shen et al. (2011)
*Lactobacillus brevis*	+(*Ca thl*, *Ca hbd*, *Ca crt*, *Td ter*)	MRS	Batch	0.817	n.a.	0.005	Li, Wu, et al. (2020)
*Pseudomonas putida*	+*(Ca thl*, *Ca hbd*, *Ca crt*, *Ca bcd*, *Ca etfAB*, *Ca adhE1*)	Glycerol	Batch	0.122	0.024	0.002	Nielsen et al. (2009)
*Saccharomyces cerevisiae*	Δ(*adh1‐6*, *sfa1*, *gpd2*, *ald6*) + (*Sc ERG10*, *Ca hbd*, *Ca crt*, *Td ter Ca adhE2*, *Ec eutE*, *Ec coaA*, *Ec adhE* ^A267T/E568K/R577S^, *Sc FMS1*)	Glucose	Batch	0.859	0.071	0.086	Schadeweg and Boles (2016)

*Note*: The symbols ∆ and + precede the name of the genes that were disrupted or overexpressed, respectively. The acronym next to the gene name refers to the microbial source of that gene.

Abbreviations: *Aa*, *arxula adeninivorans*; *adh1‐6*, *adhE*, alcohol dehydrogenase; *ald6*, acetaldehyde dehydrogenase; *amyE*, α‐amylase; *Cb*, *Candida boidinii*; *coaA*, pantothenate kinase; *cop*, acyl‐CoA oxidase 3; *cpp*, acyl‐carnitine permease 1; *ctp*, acyl‐canitine transferase 2; *ERG10*, thiolase; *eutE*, butyraldehyde dehydrogenase; *FMS1*, amine oxidase; *frdBC*, fumarate reductase; *gpd1‐2*, glycerol‐3‐phosphate dehydrogenase; n.a., not available; *pyrD*, dihydroorotate dehydrogenase; *sfa1*, alcohol dehydrogenase*; thrC*, threonine synthase.

Application of butanol‐producing *A. adeninivorans*, *C. tyrobutyricum*, or *E. coli* to consolidated fermentation of lignocellulosic biomass requires further engineering with cellulolytic enzymes or use in artificial consortia with cellulolytic microorganisms. To date, only a little research effort has been reported in these directions. The butanol‐overproducing *C. tyrobutyricum* strain Δ*cat1*::*adhE2* (Zhang et al., 2018) was used for fermentation of paper mill sludge hydrolysate leading to the generation of 16.5 g L^−1^ butanol with yield and productivity similar to those obtained through glucose fermentation (Table 2) (Cao et al., 2020). Similar results have been reported by using other butanol‐engineered *C. tyrobutyricum* strains on a variety of (ligno)cellulosic biomass hydrolysates (e.g., cassava bagasse, corn fiber, cotton stalk, microalgae, and soybean hull), namely butanol titers, yields and productivities comprised between 12–16 g L^−1^, 0.24–0.34 g g^−1^, and 0.15–0.26 g L^−1^ h^−1^ (Fu et al., 2021; Huang et al., 2019; Li et al., 2019; Yu et al., 2015). Hopefully, the high potential of butanol‐producing *C. tyrobutyricum* will be tested in the near future in a more consolidated fermentation of lignocellulosic feedstocks. Based on the high genetic tractability of *E. coli*, a consortium of engineered *E. coli* strains was developed which was able to directly ferment ionic liquid‐treated switchgrass to butanol (Bokinsky et al., 2011). First, in *adhE*‐lacking *E. coli*, an artificial butanol pathway (consisting of the *hdb*, *crt*, *bcd*, *etfAB*, and *adhE2* genes from *C. acetobutylicum*) was introduced. Then (i) a cellulose‐hydrolyzing subpopulation was engineered by introducing heterologous endocellulase and β‐glucosidase and (ii) a hemicellulose‐depolymerizing subpopulation was developed through an additional expression of heterologous endoxylanase and xylobiosidase (Table 3). Co‐culture of these two *E. coli* strains enabled them to grow on 3.3% w/v ionic liquid‐treated switchgrass and produce 0.028 g L^−1^ butanol (Bokinsky et al., 2011). Because of the very low butanol titer obtained, this study only represents a proof of concept. However, it should be remembered that in the latter study, *E. coli* was not engineered with the high‐performing artificial butanol pathway described by Shen et al. (2011). Hence, improvement of direct cellulosic butanol production by engineered *E. coli* seems feasible. So far, no attempt to co‐culture butanol overproducing *E. coli* with (hemi)cellulolytic microorganisms has been reported. However, a consortium consisting of the cellulolytic fungus *Trichoderma reesei* and a recombinant isobutanol‐producing *E. coli* was developed which could directly ferment microcrystalline cellulose to isobutanol (Minty et al., 2013).

**TABLE 3:** Maximum production of butanol was reported for direct fermentation of lignocellulosic feedstocks by the different approaches illustrated in this review

Strategy	Strain(s)	Substrate	Fermentation mode	Titer (g L^−1^)	Yield (g g^−1^)	Productivity (g L^−1^ h^−1^)	References
Recombinant cellulolytic strategy	^a^ *Evolved Clostridium acetobutylicum +(cel48FA)*	PASC	Continuous	n.a.	n.a.	n.a.	Soucaille et al. (2010)
Native cellulolytic strategy	*Clostridium cellulovorans* Δ(*xylR*, *araR*) +(*Ca adhE1*, *Td ter*, *Ct cat1*, *Ca xylT*)	Alkali extracted corn cob	Batch	4.96	n.a.	0.05	Wen, Ledesma‐Amaro, Lu, Jin, and Yang (2020)
	*C. cellulovorans* +(*Ca adhE2*, *Ca fnr*)	Cellulose + 150 μM methyl viologen	Batch	5.74	0.36	0.01	Bao et al. (2021)
Artificial microbial consortium	^b^ *C. cellulovorans* 743B [∆(*ldh*, *ack)* i‐*hydA* +(*Cc buk*)] + *Clostridium beijerinckii* NCIMB 8052 [∆*xylR* +(*Cb xylT Cb ctfAB*)]	Alkali extracted corn cob	Fed‐batch	11.50	0.14	0.11	Wen et al. (2017)
	*Thermoanaerobacterium thermosaccharolyticum M5* + *C. acetobutylicum NJ4*	Xylan	Batch	13.28	0.26	0.08	Jiang et al. (2020)
Fusant strains	*C. beijerinckii‐Clostridium thermocellum* fusant	Dilute sulphuric acid‐pretreated wheat straw	Batch	14.13	0.29	0.12	Begum and Dahman (2015)
Engineering of unconventional microorganisms	^c^ *Escherichia coli* +(*Ca hdb*, *Ca crt*, *Ca bcd*, *Ca etfAB*, *Ca dhE2*, *Bs* endo, *Cj* beta) + *E. coli* +(*Ca hdb*, *Ca crt*, *Ca bcd*, *Ca etfAB*, *Ca adhE2*, *Cs xyn10B*, *Cj gly43F*)	Ionic liquid treated switchgrass	Batch	0.03	n.a.	n.a.	Bokinsky et al. (2011)

Abbreviations: *araR*, arabinose utilization negative regulator; n.a., not available.

^a^ *C. acetobutylicum* was engineered by replacing the native endoglucanase Cel48A with a chimeric protein consisting of the catalytic domain of *C. cellulolyticum* Cel48F and the dockerin module of *C. acetobutylicum* Cel48A. Adaptive evolution was used to improve the growth of this engineered strain on PASC.

^b^ In the engineered *C. cellulovorans* strain the hydrogenase encoding gene (*hydA*) was down‐regulated by CRISPR interference.

^c^ This consortium consists of two *adhE*‐lacking *E. coli* in which an artificial butanol pathway from *C. acetobutylicum* was introduced. Then (i) a cellulose‐hydrolyzing subpopulation was engineered by introducing a *Bacillus* sp. endocellulase (*Bs* endo) and a β‐glucosidase from *Cellvibrio japonicus* (*Cj* beta) and (ii) a hemicellulose‐depolymerizing subpopulation was developed through the additional expression of *Clostridium stercorarium* endoxylanase *xyn10B* and *C. japonicus* xylobiosidase *gly43F*.

## STRATEGIES FOR IMPROVING MICROBIAL TOLERANCE TO BUTANOL

One of the main issues of biological production of butanol is its toxicity for microbial cells, which is inherently higher than that of other established biofuels, such as ethanol, owing to its higher hydrophobicity (Heipieper et al., 2007; Wilbanks & Trinh, 2017). Butanol toxicity is mainly related to the impairment of structure and functions of biological membranes such as increase of membrane fluidity (Fletcher et al., 2016), inhibition of membrane‐bound ATPases, decrease or elimination of ΔpH and Δψ, and reduction of glucose uptake (Alsaker et al., 2010; Bowles & Ellefson, 1985; Tomas et al., 2004; Venkataramanan et al., 2015). Native butanol‐producers such as *C. acetobutylicum* generally show rather low tolerance (i.e., 1–2% v/v butanol) (Huang et al., 2010; Nicolaou et al., 2010). Cellulolytic clostridia such as *C. thermocellum* (Tian, Cervenka, et al., 2019) or *C. cellulovorans* (Costa et al., 2021; Yang et al., 2015) show even lower resistance since they cannot grow at butanol concentrations higher than 5–8 g L^−1^ (i.e., 0.6–1% v/v), respectively. Among other potential hosts for recombinant butanol production, *E. coli* growth is inhibited at 1% v/v (Si et al., 2016) while microbes tolerating the highest butanol concentrations include bacteria belonging to the *Pseudomonas* genus (2%–3% v/v) (Cuenca et al., 2016; Halan et al., 2017) and lactic acid bacteria (3.5%–4% v/v) (Li et al., 2021). Butanol toxicity limits its titer in batch fermentation, hence challenging the viability of industrial butanol production processes. For this reason, substantial interest has been addressed in identifying the genetic determinants involved in butanol tolerance and generating butanol hypertolerant strains (Arsov et al., 2021).

**FIGURE 4:**
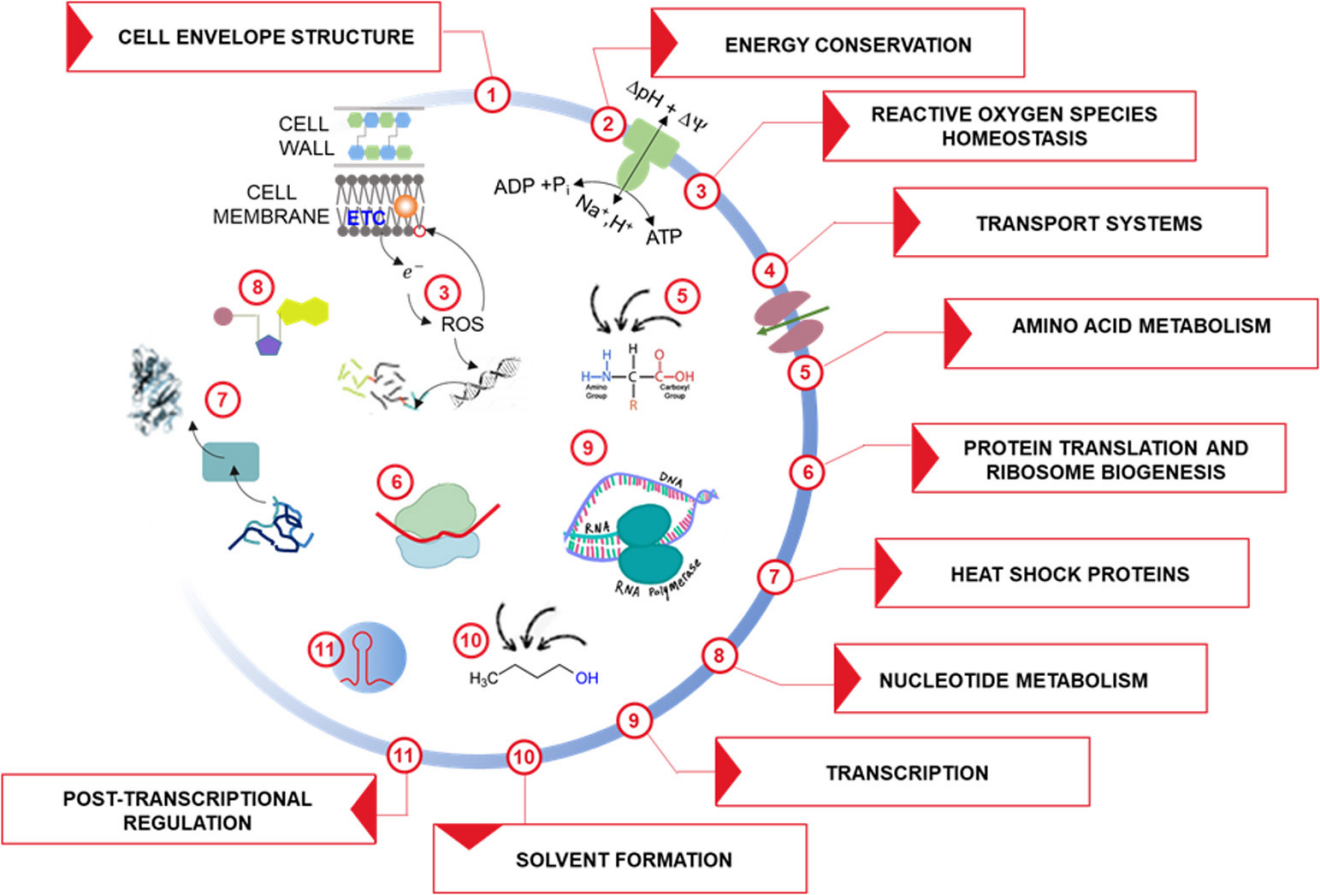
Butanol response mechanisms. The main cell response mechanisms mediating tolerance to butanol challenge are displayed. Depicted mechanisms are shortly discussed in the main text or referenced to relevant studies provided in Table S1. Abbreviations: ETC, electron transfer chain.

The butanol stress response has been investigated in several microbial species, including native butanol producers (Alsaker et al., 2010; Sedlar et al., 2019; Venkataramanan et al., 2015) and non‐producing microorganisms such as lactic acid bacteria (Liu et al., 2021; Petrov et al., 2021), *P. putida* (Cuenca et al., 2016), *E. coli* (Rutherford et al., 2010), *C. cellulovorans* (Costa et al., 2021), and *Synechocystis* sp. (Tian et al., 2013). These studies consistently depicted the involvement of a very complex network of mechanisms (Figure 4, Table S1) (Arsov et al., 2021). The most established and ubiquitous cell responses to butanol exposure include (i) activation of the homeoviscous adaptation (namely, a modification of the cell membrane composition to cope with the increased fluidity caused by solvents); (ii) overexpression of heat shock proteins (HSPs, e.g., GroESL, DnaKJ, Hsp90, ClpC, and HtrA) and downregulation of protein translation (to attenuate the effects of butanol on protein denaturation); and (iii) adaptation of biochemical systems for pH and energy homeostasis. However, a more detailed analysis reveals a number of gaps in understanding the mechanisms underlying these observations (such as in the events leading to the downregulation of ribosome activity) or inconsistencies between one microbial model to another. For instance, butanol exposure was reported to downregulate the expression of ribosomal proteins in most microorganisms investigated so far (Alsaker et al., 2010; Fu et al., 2013; Sedlar et al., 2019; Tian et al., 2013; Venkataramanan et al., 2015), whereas an opposite trend has recently been observed in *C. cellulovorans* (Costa et al., 2021). Some evidence of post‐transcriptional regulation of the expression of these genes and/or use of alternative translation machinery under solvent stress has been reported (Venkataramanan et al., 2015) but further confirmation is required. Although differential expression of ATPases has frequently been observed in butanol‐challenged microorganisms (which has been related to maintenance of energy/pH homeostasis), this may consist in either up‐ (Costa et al., 2021; Ghiaci et al., 2013) or down‐regulation (Fu et al., 2013; Liu et al., 2021) depending on the strain. Some inconsistencies are likely related to the fact that different microorganisms, depending on their gene repertoire, may employ alternative mechanisms to address the same issue. This seems to apply to strategies to adjust membrane fluidity which may involve changes in the content of saturated and/or cis/trans unsaturated (Bernal et al., 2007; Huffer et al., 2011) and/or cyclopropanated (Kolek et al., 2015) and/or branched‐chain (Mansilla et al., 2004) fatty acids depending on the microorganism. The need for more in‐depth understanding is even more evident in other metabolic pathways affected by butanol challenges such as amino acid and nucleotide metabolism (Alsaker et al., 2010; Costa et al., 2021). Therefore, a global detailed comprehension of microbial response to butanol stress that could be used to rationally improve butanol tolerance currently remains elusive.

In such a complex framework, it is not surprising that improvement of butanol tolerance through targeted gene manipulation has so far achieved only limited results (Table 4). These strategies have generally been based on the modification of single/few genes that affect cellular structures/functions (e.g., membrane composition, membrane transport, and adaptation to oxidative stress). Overexpression of protein chaperones is among the most frequently used approaches (Table 4). It is worth noting that overexpression of some chaperones such as *groESL* and *grpE* resulted in an increase in butanol tolerance in different microbial strains (Mann et al., 2012; Tomas et al., 2003; Zingaro & Papoutsakis, 2012), while upregulation of other chaperones (e.g., DnaJ, IbpA, and IbpB) was not effective (Zingaro & Papoutsakis, 2012). The expression of chaperones from extremophiles has been reported to confer higher butanol tolerance with respect to their mesophilic counterparts (Liao et al., 2017). Some studies combined targeted and random strategies by performing random mutagenesis on selected gene targets, that is, the cyclic AMP receptor protein (*crp*) (Zhang et al., 2012), the σ^70^ RNA polymerase subunit (Si et al., 2016), or the *secB* chaperon (Xu et al., 2019). However, all these studies (mainly performed on *C. acetobutylicum* or *E. coli*) have so far failed to generate strains that can tolerate higher than 2% (v/v) butanol. From this standpoint, random approaches (e.g., random mutagenesis, genome shuffling, and adaptive evolutionary engineering) proved to be more successful since mutant *C. acetobutylicum* strains able to tolerate up to 3%–4% (v/v) butanol were reported (Liu et al., 2012, 2013) (Table 5). Strains developed by these strategies typically feature mutations related to multiple cell structures (e.g., cell membrane and cell wall) and functions (e.g., membrane transport, gene transcription, and protein biosynthesis). Although time consuming, random approaches currently seem more effective in selecting multiple‐gene trait combinations leading to higher butanol resistance. Irrespective of the method (targeted, random) used to enhance butanol tolerance, most of the improved strains (equipped with a butanol pathway) actually showed higher butanol production as well (Tables 4 and 5) which encourages future research in this direction.

**TABLE 4:** Improvement of butanol tolerance by targeted metabolic engineering

Biological framework	Gene modification	Strain	Effect on butanol tolerance	Maximum tolerated butanol (% v/v) [increase^a^]	Effect on butanol titer^a^	References
Cell envelope structure	+ *Ca cfa*	*C. acetobutylicum* ATCC 824	≈75% higher biomass production after 100 min at ≈0.8% (v/v) butanol	0.82 [0]	≈80% lower	Zhao et al. (2003)
	+ FAS genes	*E. coli* MDB5	≈40–300% higher biomass production at 0.5–1.5% (v/v) butanol	2 [0]	–	Bui et al. (2015)
	+ *Pa cti*	*E. coli* MG1655	16% increased specific growth rate at 0.6% (v/v) butanol	0.6 [0]	–	Tan et al. (2016)
ROS homeostasis	+ *Ec gshAB*	*C. acetobutylicum* DSM1731	Survival at 2.3% (v/v) butanol was > 33% longer	2.04 [+65%]	10%–37% higher	Zhu et al. (2011)
	+ membrane‐targeted *Om/Mm* metallothionein	*E. coli* BL21	Butanol tolerance increased from 0.5% to 1.5% (v/v)	1.5 [+201%]	–	Chin et al. (2013)
Transport	+*Ec feoA*	*E. coli* MDB5	Up to 21% higher biomass production at 1% (v/v) butanol	2 [0]	–	Bui et al. (2015)
	+*Pp srpB*	*C. saccharoperbutylacetonicum* N1‐4	20‐30% higher growth at 0.8‐1.2 % (v/v) butanol	1.2 [0]	≈20% lower	Jiménez‐Bonilla et al. (2020)
	+*Ca btrTM*	*C. acetobutylicum* ATCC 824	46.5% increased tolerance at 1% (v/v) butanol	1 [0]	(faster growth and butanol production)	Yang et al. (2020)
Heat shock proteins	+*Ca groESL*	*C. acetobutylicum* ATCC 824	Growth inhibition by 0.25, 0.50, and 0.75% (v/v) butanol was reduced by 85, 40 and 50%, respectively.	0.75 [0]	≈30% higher	Tomas et al. (2003)
	+*Ca groESL* or *Ca grpE* or *Ca htpG*	*C. acetobutylicum* ATCC 824	Improved survival after 2 h at 2% (v/v) butanol: +*groESL*, 45%; +*grpE*, 25%; +*htpG*, 56% (wt strain, no survival)	2 [+100%]^b^	+*groESL*, 30% higher +*grpE*, 49% lower +*htpG*, 32% lower	Mann et al. (2012)
	+(*Ec clpB*, *Ec groESL*)	*E. coli* MG1655	78% increase in c.f.u. after 24 h at 1% butanol	1 [0]	–	Zingaro and Papoutsakis (2012)
	+(*Ec clpB*, *Ec groESL*, *Ec grpE*)	*E. coli* MG1655	390% increase in c.f.u. after 24 h at 1% butanol	1 [0]	–	Zingaro and Papoutsakis (2012)
	+*Ca groESL* or *Dw groESL* or *Dw dnaK*	*C. acetobutylicum* ATCC 824	Increased growth rate at 1% (v/v) butanol: +*Ca groESL*, 25%; +*Dw groESL*, 35%; +*Dw dnaK*, 45%	1 [0]	+*Dw dnaK*, 49.4% higher +*Dw groESL*, 28.7% higher +*Ca groESL* 23.0% higher	Liao et al. (2017)
	+*Ec secB*_*T10A*_	*E. coli* JM109	Maximum tolerated butanol concentration was increased from 1.4 to 1.8% (v/v)	1.8 [+50%]	–	Xu et al. (2019)
Transcription	+*Ca spo0A*	*C. acetobutylicum* ATCC 824	Prolonged glucose utilization (from 20 to 70 min) at 0.2 and 0.6% (v/v) butanol	0.6 [0]	≈200% higher	Alsaker et al. (2004); Gao et al. (2020)
	mutant *Ec crp*	*E. coli DH5α*	2‐fold higher growth rate at 1.2 % (v/v) butanol	1.2 [0]	–	Zhang et al. (2012)
	mutant *Ec* σ^70^	*E. coli* JM109	Maximum tolerated butanol concentration was increased from 1.2 to 2% (v/v)	2 [+67%]	–	Si et al. (2016)
Signal transduction	∆cac3319	*C. acetobutylicum* ATCC 55025	Maximum tolerated butanol concentration was increased from 1.48% to 1.73% (v/v)	1.73 [+40%]	≈44% higher (90% higher productivity)	Xu, Huang, et al. (2015); Xu, Zhao, et al. (2015)
Post‐transcriptional regulation	+*Ca tm sRNA*	*C. acetobutylicum* ATCC 824	8–257‐fold higher survival after 48 h at 1–2% (v/v) butanol	2 [0]	–	Jones et al. (2016)
	+*Ca 6s sRNA*	*C. acetobutylicum* ATCC 824	22‐fold higher survival after 24 h at 2% (v/v) butanol; 8‐14 fold higher survival after 48 h at 1%–1.5% (v/v) butanol	2 [0]	20% higher	Jones et al. (2016)

*Note*: The symbols ∆ and + precede the name of the genes that were disrupted or overexpressed, respectively. The acronym next to the gene name refers to the microbial source of that gene.

Abbreviations: *btrTM*, putative ABC transporter; cac3319, histidine kinase; *cfa*, cyclopropane fatty acid synthase; c.f.u., colony forming unit; *crp*, cyclic AMP receptor protein; *cti*, periplasmic cis‐trans isomerase; *Dw*, *Deinococcus wulumuqiensis*; FAS, fatty acid synthesis; *feoA*, iron uptake protein; *gshAB*, γ‐glutamylcysteine synthetase and glutathione synthetase; *Om*, *Oreochromis mossambicus* (tilapia fish); *Mm*, *Mus musculus* (mouse); *Pa*, *Pseudomonas aeruginosa; Pp*, *Pseudomonas putida*; *secB*_*T10A*_, mutant chaperon SecB; *srpB*, subunit of the SrpABC efflux pump; σ^70^, RNA polymerase subunit.

^a^ Comparison with the parental strain.

^b^ After 4 h exposure at butanol 2% (v/v).

**TABLE 5:** Representative studies exploiting untargeted approaches to enhance the tolerance of strains to butanol challenge

Approach	Parental strain	Key genetic features	Maximum tolerated butanol (% v/v) [increase^a]^	Effect on butanol titer^a^	References
Adaptive laboratory evolution	*C. acetobutylicum* ATCC 55025	Key mutant genes include *ftsY* (membrane protein synthesis), cac3319 (orphan histidine kinase), efflux pumps, and genes involved in biosynthesis and metabolism of phospholipids, peptidoglycan, sporulation, and stress adaptation.	1.98 [+33%]	≈70% higher	Yang and Zhao (2011); Xu, Zhao, et al. (2017); Xu, Li, et al. (2017)
	*C. thermocellum*	Improved butanol tolerance is mainly related to *adhE* ^D494G^ mutation	1.85 [+200%]	–	Tian, Cervenka, et al. (2019); Tian, Conway, et al. (2019)
	*C. cellulovorans* DSM 743B +(*Ca adhE1*, *Ca ctfAB*, *Ca adc*)	n.a.	1.48 [+33%]	50.5% higher^b^	Wen et al. (2019)
Artificial simulation of bio‐evolution	*C. acetobutylicum* D64	n.a.	4 [+100%]	25% higher	Liu et al. (2013)
Genomic library enrichment	*C. acetobutylicum* ATCC 824	Genes imparting solvent tolerance include: CAC0003 (unknown function) and CAC1869 (transcriptional regulator involved in phase transition)	1.56 [+20%]	n.a.	Borden and Papoutsakis (2007)
	*E. coli* K‐12 strain BW25113	Gene modifications conferring the largest improvement in butanol tolerance: +*entC* and +*feoA* (both involved in iron transport/metabolism) and ∆*astE* (glutamate metabolism)	1.70 [+245%]	–	Reyes et al. (2011)
Genome shuffling	*C. acetobutylicum* DSM 1731	Genomic analysis identified two insertion sites, four deletion sites, and 67 SNVs affecting transport, cell structure, DNA replication, and protein translation. Altered phase‐associated expression of proteins.	2.35 [+58%]	23% higher	Mao et al. (2010); Bao et al. (2014)
^12^C^6+^ irradiation	*C. acetobutylicum* ATCC 824	Mutant genes feature cell membrane functions (transport, signal transduction, cell wall, and cell membrane synthesis)	2 [+100%]	≈40% higher	Gao et al. (2021)
Nitrogen ion beam implantation	*C. acetobutylicum* D64	n.a.	3 [+50%]	13–20% higher	Liu et al. (2012)

Abbreviations: *adhE*, bifunctional alcohol‐aldehyde dehydrogenase; n.a., not available; SNV, single nucleotide variation.

^a^ Comparison with the parental strain.

^b^ These data refer to evolved *C. cellulovorans* DSM 743B +(*Ca adhE1*, *Ca ctfAB*, and *Ca adc*) with respect to non‐evolved *C. cellulovorans* DSM 743B +(*Ca adhE1*, *Ca ctfAB*, and *Ca adc*).

It is clear that adaptation to solvents involves the whole microbial cell, similar to responses to other major physical‐chemical stresses (e.g., heat shock and pH) (Mazzoli, 2021). The availability of genome‐wide engineering techniques such as multiplex automated genome engineering could suit these complex gene modifications (Si et al., 2017). Alternatively, strategies targeting global gene regulators involved in stress response could potentially better exploit the native regulatory networks evolved by microorganisms to face these growth conditions (Jones et al., 2016; Xu et al., 2021). For instance, a number of recent reports indicated an important role of small non‐coding RNAs (sRNAs) and RNA chaperones (e.g., Hfq) in tolerance to a variety of stresses, including butanol, in different microorganisms (Costa et al., 2021; Jones et al., 2016; Sun et al., 2017; Venkataramanan et al., 2013). It is likely that global response to solvent stress may be under the control of general regulatory control system(s), which could be engineered to provide more efficient adaptation to this stressful condition.

## CONCLUSIONS

The renewed substantial interest in the biological production of butanol has inspired a variety of strategies aimed at developing CBP of lignocellulosic biomass to this solvent. The development of artificial consortia (Jiang et al., 2020; Wen et al., 2017) and fusant strains (Begum & Dahman, 2015) have so far achieved the highest butanol titer and productivity (Table 3). A cautious estimation of fusant potential for cellulosic butanol production currently seems recommended based on the very limited number of studies (which used partially hydrolyzed feedstocks) and general issues of protoplast fusion approaches (e.g., low genetic stability of fusants). Co‐culture‐based fermentations are still relatively complex and long owing to the different conditions (i.e., pH and/or temperature) required for (hemi)cellulolytic and butanol‐producing strains (Table 1), which significantly limit their efficiency compared to traditional ABE fermentation (Abo et al., 2019; Gu et al., 2011). Model‐driven analysis (Salimi et al., 2010) and/or use of engineered strains (Wen et al., 2017; Wen, Ledesma‐Amaro, Lu, Jiang, et al., 2020) may help develop more robust and synergistic consortia. Significant progress but also important issues have been reported as regards both NCS and RCS. RCS seems inherently more challenging owing to the complexity of native cellulase systems and issues in heterologous expression of cellulases which so far resulted in very few recombinant strains able to ferment crystalline cellulose (Anandharaj et al., 2020). NCS is at an earlier stage and seem to have a larger repertoire of metabolic levers. Towards this direction, the improvement of genetic tools for *C. cellulolyticum* and *C. cellulovorans* seems necessary. Moreover, it is worth extending NCS to additional cellulolytic paradigms as soon as gene manipulation tools are developed (e.g., *M. thermophila*). Butanol titers achieved by some alternative recombinant butanol producers (e.g., *C. tyrobutyricum* and *E. coli*) are among the highest reported so far. It is desirable that the potential of these strains is tested in the near future in CBP of lignocellulosic biomass for instance by co‐culture with (hemi)cellulolytic microbes. Based on this diverse repertoire of approaches and parallel advances in strategies aimed to improve butanol tolerance, we are confident of further progress in the development of lignocellulose CBP to butanol that could contribute to an environmentally sustainable economy.

## CONFLICT OF INTEREST

The authors declare no conflict of interest.

## Supporting information


Appendix S1:
