# Supplementary material for: Current progress on engineering microbial strains and consortia for production of cellulosic butanol through consolidated bioprocessing
Source: Microb Biotechnol. 2022 Sep 27;16(2):238–61. doi: 10.1111/1751-7915.14148 (PMC9871528; doi:10.1111/1751-7915.14148)
Supplement: Supplementary file 1 — Appendix S1: [file MBT2-16-238-s001.docx]

Table S1. Butanol tolerance in several microbial model organisms. The table shows the objective and the methodology adopted in each study, along with a short overview of the experimental factors. The abbreviation “n.a.” stands for “not available” data whenever any experimental detail is not made explicit in the reference manuscript. For each study, the table reports whether the study made use of any omics approach and/or metabolic engineering technique. The abbreviations used to describe the omics approaches follow: GC-MS stands for Gas Chromatography-Mass Spectrometry (GC-MS),  2D-LC-MS/MS for two-Dimensional Liquid Chromatography coupled to [tandem Mass Spectrometry](https://www.sciencedirect.com/topics/biochemistry-genetics-and-molecular-biology/tandem-mass-spectrometry), MALDI-TOF-MS for Matrix-Assisted Laser Desorption/Ionization- Time-Of-Flight Mass Spectrometry, MALDI-TOF/TOF for Matrix-Assisted Laser Desorption/Ionization Time-Of-Flight/Time-Of-Flight, SNP stands for Single Nucleotiode Polymorphism, InDel for Insertion or Deletion of bases in the genome, iTRAQ stands for Isobaric Tag for Relative and Absolute Quantitation.

| Strain | Metabolic engineering | Omics assay | Objective | Methodology | Maximum tolerated concentration (g L^-1^) | Experimental factors | Reference |
| --- | --- | --- | --- | --- | --- | --- | --- |
| *Clostridium acetobutylicum* ATCC 824; mutant SA-1 | NO | Any | butanol tolerance | Setup: serial enrichment procedure to develop butanol tolerant strain; Benefit: development and characterization of a butanol tolerant strain | 18.6 | Stressor: 0 to 20 g L^-1^ of butanol; OD: 0.8; Volume: 9 ml; Agitation: any; pH: n.a. | (Lin and Blaschek, 1983) |
| *C. acetobutylicum* ATCC 824 | NO | Any | short-term (<2h) butanol-induced stress response | Setup: characterization of cellular physiology upon butanol challenge; Benefit: exploration of the effects of butanol on membrane properties | 8.10 | Stressor: 0%, 1%, 1.5% (v/v) butanol; OD: mid-log stationary; Volume:; Agitation: any; pH: n.a. | (Vollherbst Schneck *et al.*, 1984) |
| *C. acetobutylicum* ATCC 824; mutant SA-2 | NO | Any | short-term (<2h) butanol-induced stress response and butanol tolerance | Setup: comparative analysis of the effects of butanol challenge on membrane fluidity and fatty acid composition between wild-type and butanol tolerant mutant strains; Benefit: exploration of cellular mechanisms underlying butanol tolerance | 12.1 | Stressor: 0%, 1%, 1.5% (v/v) butanol; OD: n.a.; Volume: 1.8 L; Agitation: any: pH: n.a. | (Baer *et al.*, 1987) |
| *C. acetobutylicum* ATCC 824 | NO | cDNA microarray analysis | short-term (<2h) butanol-induced stress response | Setup: transcriptome analysis of response to acetate, butyrate and butanol stresses; Benefit: disambiguation of common stress responses from stress-specific responses | 3.71 | Stressor: 50 mM butanol; OD: 0.6; Volume: 1 L: Agitation: any; pH: n.a. | (Alsaker *et al.*, 2010) |
| *C. acetobutylicum* 824(pMSPOA) (a spo0A-overexpressing *C. acetobutylicum* strain with enhanced sporulation); *C. acetobutylicum* SKO1 (spo0A knockout neither sporulating nor producing solvents) ; plasmid control strain | YES | cDNA microarray analysis | short-term (<24h) butanol-induced (0.6% v/v) stress response | Setup: transcriptome analysis of butanol-induced stress response in various strains differing for the expression level of spo0A; Benefit: assess the interelationship between sporulation and strain tolerance in response to solvent stress | 4.86 | Stressor: 0.2%, 0.4%, 0.6% (v/v) butanol; OD: 0.6-0.7; Volume: 200 ml; Agitation: any; pH: n.a. | (Alsaker *et al.*, 2004) |
| *C. acetobutylicum* Rh8 | YES | Genome sequencing analysis | butanol tolerance | Setup: Comparative genomic and proteomic analyses of the butanol-tolerant strain and its parental strain; Benefit: individuating genomic changes associated with the apperance of butanol tolerance and study the interrelationship between genomic and proteomic changes; | 19.0 | Stressor: not applied; OD: not applied; Volume: not applied; Agitation: not applied; pH: not applied | (Bao *et al.*, 2014) |
| *Clostridium cellulovorans* (83151-*adhE2*) | YES | Any | butanol-induced stress response | Setup: genetic engineering of cellulolytic bacterium for butanol production; Benefit: production of butanol from cellulosic biomass of enhanced efficiency owing to larger cellulosome repertoire and enhanced selectivity in the absence of acetone production | 8.00 | Stressor: 0, 2, 4, 6, 8, 10 g L^-1^ butanol; OD: 1; Volume: 50 ml; Agitation: any; pH: 6.0-7.0 | (Yang *et al.*, 2015) |
| *C. acetobutylicum* 824 | NO | GM-MS analysis | stress response at 8h (acidogenesis) and 24 h (solventogonesis) after butanol addition | Setup: analysis of butanol stress-associated intracellular biochemical changes at acidogenesis and solventogenesis phases; Benefit: gain insights into metabolites' changes upon butanol stress | 12.2 | Stressor: 0.5%, 1.0%, 1.5%, 2.0%, 2.5% (v/v) butanol; OD: 0.1; Volume: 100 ml; Agitation: any; pH: n.a. | (Wang *et al.*, 2016) |
| *C. cellulovorans* DSM 743B and ZQW3 | YES | Any | increase in butanol production and tolerance | Setup: combination of evolutionary engineering with metabolic engineering; Benefit: combination of both cellulolytic and butanol-producing phenotypes within a single microorganism | 12.0 | Stressor: 0, 3, 6, 9, 12 g L^-1^ butanol; OD: 1; Volume: 10 ml; Agitation: any; pH: n.a. | (Wen *et al.*, 2019) |
| *C. acetobutylicum* strain 824(pGROE1) overexpressing groESL; plasmid control strain 824(pSOS95del) | YES | cDNA microarray analysis | long-term (>24 h) response of butanol stress (plasmid control strain 824(pSOS95del) and butanol tolerance (strain overexpressing groESL) | Setup: generation, physiological characterization and transcriptome analysis of a butanol tolerant strain in comparison to the plasmid control strain; Benefit: disambiguation of the changes associated with the response of, respectively, the butanol tolerance and butanol stress | 6.07 | Stressor: 0.25%, 0.75% (v/v) butanol; OD: 0.8; Volume: 200 ml; Agitation: any; pH: n.a. | (Tomas *et al.*, 2004) |
| *C. acetobutylicum* ATCC 824 | NO | cDNA microarray analysis | short-term (< 2h) butanol stress response | Setup: comparative analysis of butanol versus butyrate stress responses; Benefit: understanding the common stress response as well as the specialized, stressor-dependent responses | 6.67 | Stressor: 30, 60, 90 mM butanol; 30, 40, 50 mM butyrate; OD: 1; Volume: n.a.; Agitation = n.a.; pH: n.a. | (Wang *et al.*, 2013) |
| *C. acetobutylicum* ATCC 824 | NO | cDNA microarray analysis, RNAseq analysis, iTRAQ-based 2D-LC-MS/MS data analayis | short-term butanol stress response | Setup: comparative transcriptome/proteome analysis of butanol/butyrate stress response at multiple post-stress time points; Benefit: disambiguation of stressor-dependent changes and exploration of complex expression patterns | 6.67 | Stressor: 0, 30, 40, 50 mM butyrate; 0, 30, 60, 90 mM of butanol; OD: 1.0; Volume: 4 L; Agitation: n.a.; pH: >5 | (Venkataramanan *et al.*, 2015) |
| *C. acetobutylicum* ATCC 824 | YES | cDNA microarray analysis | short-term butanol stress response | Setup: comparison between the responses to low/medium/high butanol stresses of a wild-type and a modified strain; Benefit: assess the impact of targeted gene overexpression on solvent tolerance | 6.07 | Stressor: 0.25%, 0.50%, and 0.75% (v/v) butanol; OD: 1; Volume = 80 ml; Agitation: any; pH: n.a. | (Tomas *et al.*, 2003) |
| *C. acetobutylicum* DSM1731 (butanol tolerance 13 g L^-1^); modified *C. acetobutylicum* Rh8 (butanol tolerance 19 g L^-1^). | YES | MALDI-TOF MS and MS/MS analysis | long-term butanol stress response in acidogenic and solventogenic phases | Setup: comparative analysis of a wild-type strain and its mutant Rh8 (with increased butanol tolerance/yield) in acidogenesis/solventogenis phases; Benefit: elucidation of the relationship between the mechanisms underlying butanol tolerance and yield | 18.0 | Stressor: 0, 6, 12, 18 g L^-1^ butanol; OD: 1; Volume: 100 ml; Agitation: any; pH: n.a. | (Mao *et al.*, 2010) |
| *C. acetobylicum* ATCC 824; 824(pCAC1869), 824(pCAC0003) | YES | DNA microarray | short-term butanol stress and butanol tolerance | Setup: genomic library enrichment with library inserts selected using *i)* single round of challenges at discrete butanol stress levels, or *ii)* serial transfer of stationary-phase cultures into progressively higher butanol concentration; Benefit; identification of genomic inserts containing genes conferring butanol tolerance under conditions of selective growth | 9.72 | *Approach I*: Stressor: 0, 0.6, 1.2, 1.5% (v/v) butanol; OD: 0.6; Volume: 450 ml; Agitation: any; pH: n.a.; *Approach II*: Stressor: 0%, 0.6%, 1.2%, 1.5% (v/v) butanol; OD: stationary-phase transfers; Volume: 100 ml; Agitation: any; pH: n.a. | (Borden and Papoutsakis, 2007) |
| *C. acetobutylicum* D64; *C. acetobutylicum* mutant NT642 | YES | Any | butanol tolerance | Setup: random mutagenesis by nitrogen ion beam implantation; Benefit: low and controllable damage rate, higher mutation rate, and wider mutation spectrum compared to traditional mutation methods | 24.3 | Stressor: 0%, 1%, 1.5%, 2%, 2.5%, 3% (v/v) butanol; OD: 0.01; Volume: 20 ml; Agitation: any: pH: n.a. | (Liu *et al.*, 2012) |
| *C. acetobutylicum* D64; mutant T64 | YES | Any | butanol tolerance | Setup: combination of adaptive evolution and artificial selection under increasing butanol concentration (artificial simulation of bio-evolution); Benefit: enhancement of butanol tolerance and production | 32.4 | Stressor: 0%, 0.5%, 1%, 1.5%, 2%, 2.5%, 3%, 3.5%, 4% butanol; OD: n.a.; Volume: test tube (20 ml) or agar plate; Agitation: any; pH: n.a. | (Liu *et al.*, 2013a, 2013b) |
| *C. acetobutylicum* ATCC 4259 | NO | Any | butanol stress response | Setup: physiological study on the effects of exogenously added butanol in exponential phase on ATP levels, internal pH and glucose uptake; Benefit: elucidation of the physiological effects of strain exposure to butanol stress | 14.5 | Stressor: 0, 139, 167, 181, 195, 208, 222, 236, 250 mM butanol; OD: exponential phase; Volume: 100 ml; Agitation: any; pH: n..a | (Bowles and Ellefson, 1985) |
| *C. acetobutylicum* ATCC 55025; mutant *C. acetobutylicum* HKKO | YES | Any | butanol stress response | Setup: comparative physiological study of asporogenic *Clostridium* strain and its mutant featuring histidine kinase knockout; Benefit: improvement in butanol tolerance and production by elimination of the inhibitory effect due to the histidine kinase; simple costruction and absence of heterologous plasmid; strain stability | 14.0 | Stressor: 0, 8, 10, 12, 14, 16, 18 g L^-1^ butanol; OD: n.a.; Volume = 10 ml; Agitation: any; pH: n.a | (Xu *et al.*, 2015) |
| *C. acetobutylicum* ATCC 55025; mutant *C. acetobutylicum* JB200; *C. acetobutylicum* ATCC 824; | YES | SNP and InDel analysis, and capillary LC-MS/MS | butanol tolerance | Setup: adaptive evolution engineering; comparative protein analysis between the butanol tolerant mutant JB200 and its asporogenic parental strain 55025 at acidogenesis and late stationary stage; genomic analysis (SNPs, InDels) of JB220 and 55025 with the reference strain *C. acetobutylicum* ATCC 824; Benefit: generation and characterization of strain endowed with increased butanol tolerance | 16 | B*utanol adaptation:* Stressor: intermittent challenge with increasing butanol concentration self-produced in the FBB reactor;Volume: 2L; Agitation = 100 rpm; pH: >5; OD: exponential phase; *Butanol tolerance assessment*: Stressor: 0, 8, 10, 12, 14, 16, 18 g L^-1^ butanol; OD: n.a.; Volume: 10 ml; Agitation: any; pH: n..a | (Yang and Zhao, 2011) |
| *C. acetobutylicum* ATCC 824 | NO | RNA sequencing analysis | short-term butanol stress response (15, 30, 60, 75 min post-stress) | Setup: Transcriptome analysis of a *Clostridium* culture short term response to the challenge with three butanol or butyrate concentrations; Benefit: disambiguation of stressor-dependent changes in sRNAs | 6.67 | Stressor: 30, 60, 90 mM butanol; 30, 40, 50 mM butyrate; OD: 1; Volume: 4 L; pH: >5 | (Venkataramanan *et al.*, 2013) |
| *C. acetobutylicum* ATCC 824; DDC14; DC93; DC94 | YES | MALDI-TOF/TOF MS analysis | butanol tolerance | Setup: analysis of the cytoplasmic proteomes of the overexpression strain 1731(p1518-1519), the deletion mutant DDC14, and their respective controls, in the absence or presence of 1% butanol stress; Benefit: identification of potential candidate genes to engineer butanol tolerance | 8.10 | Stressor: 0%, 1% (v/v) butanol; OD: 0.75; Volume: 100 ml; Agitation: any; pH: n.a. | (Jia *et al.*, 2012) |
| *C. acetobutylicum* ATCC 55025; ATCC 824; | NO | Genome sequence analysis, SNP and InDel analysis | butanol tolerance | Setup: genomic analysis (SNPs, InDels) of 55025 with the reference strain *C. acetobutylicum* ATCC 824; Benefit: genome-wide information valuable to egineer butanol tolerance | 12 | Stressor: 0, 8, 10, 12, 14, 16, 18 g L^-1^ butanol; OD: n.a.; Volume: 10 ml; Agitation: any; pH: n..a | (Xu *et al.*, 2017) |
| *C. acetobutylicum* ATCC 824 | NO | cDNA microarray analysis | short- and long-term butanol stress response | Setup: application of butanol pulse to cells in an acidogenic chemostat culture; transcriptome analysis of butanol-stressed cells during continuous wash-out of butanol at the sampling points 0.25h (97 mM), 1h (93 mM), 2h (88 mM), 4h (73 mM), 24h (16 mM), 48h 84mM); Benefit: isolation of transcriptionl response exclusively in response to exogenous butanol | 7.41 | Stressor: 100 mM butanol; OD: chemostat at D = 0.075 h^-1^; Volume: 1.5 L; Agitation: n.a.: pH: 5.7 | (Janssen *et al.*, 2012) |
| *C. acetobutylicum* ATCC 824 | NO | cDNA microarray analysis | short- (0.25, 1h) and long- (66 h) term stress response | Setup: acidogenic chemostat culture subject to stepwise increase in butanol concentration and samplings at 0.25, 1 and 66 h post-stress; Benefit: study of the specific butanol response with a minimum of background butanol production and uncoupled from solventogenic events that occur at lower pH values | 8.16 | Stressor = 0.25% (27.8 mM), 0.5% (58.7 mM), 0.75% (88.7 mM), 1% (110.1 mM) (v/v) butanol; OD: chemostat at 0.075 h^−1^; Volume: 1.5 L; pH: 6 | (Schwarz *et al.*, 2012) |
| *Clostridium beijerinckii* NRRL B-598 and nine butanol-tolerant mutants | NO | SNPs and InDels analysis | butanol tolerance | Setup: random chemical mutagenesis by multiple mutagenic agents: Benefit: butanol tolerant strain development | 14.9 - 24.9 | Stressor: not applied; OD: not applied; Volume: not applied; Agitation: not applied; pH: not applied | (Vasylkivska *et al.*, 2020) |
| *Lactobacillus brevis* ATCC367 | NO | cDNA microarray analysis | butanol stress response | Setup: transcriptome anaysis at increasing butanol content in growth condition; Benefit: elucidation of butanol stress response | 16.2 | Stressor: 0%, 1%, 2% (v/v) butanol; OD: 0.4; Volume: 125 ml; Agitation: 100 rpm; pH: n.a. | (Winkler and Kao, 2011) |
| *Escherichia coli* DH1 | NO | cDNA microarray analysis, two-dimensional LC-MS/MS | short-term butanol stress response | Setup: combined transcriptome and proteome analysis of butanol challenged samples; Benefit: identification of genetic programmes responsible of butanol stress response | 6.48 | Stressor: 0.8% (v/v) butanol; OD: 0.4; Volume: 300 ml; Agitation: 200 rpm; pH: n.a. | (Rutherford *et al.*, 2010) |
| Solvent-tolerant *Pseudomonas putida* DOT-T1E, S12, *Pseudomonas sp.* strain VLB120, and solvent-sensitive *P. putida* KT244 | NO | 13C-labeled tracer-based flux analysis | butanol tolerance | Setup: comparative metabolic analysis between solvent-tolerant and solvent-sensitive strains; Benefit: rationalization of the carbon flux distribution of adapted/non-adapted strains challenged by butanol stess | 48.6 | Stressor: 0%, 0.5%, 1%, 2%, 3%, 5%, 6% (volvol) butanol; OD: mid-exponential phase ; Volume: 96-well microtiter plate; Agitation: 300 rpm; pH: n.a. | (Rühl *et al.*, 2009) |
| *Lactiplantibacillus plantarum* Ym1 and 8-1 | NO | RNA sequencing analysis | butanol stress response | Setup: identification of differentially expressed genes by comparing (i) Ym1 grown in medium containing 2% (v/v) butanol to Ym1 grown in control medium; and (ii) 8-1 grown in medium with 2% (v/v) butanol, to 8-1 grown in control medium; Benefit: (i) isolation of species-specific genetic determinants of butanol stress response, and (ii) strain-specific mechanisms responsible for augmented butanol tolerance | 24.3 | Stressor: 1%, 1.5%, 2%, 2.5%, 3% (v/v) butanol; OD: 2; Volume: 250 ml; Agitation: 100 rpm; pH: n.a. | (Petrov *et al.*, 2021) |
| *Lactobacillus mucosae* BR0713–33 | NO | two dimensional LC–MS/MS | long-term (8h after butanol challenge) butanol stress response | Setup: identification of differentially expressed proteins by comparing protein profiles (i) in 0% vs 2% (v/v) butanol, (ii), 0% vs 3% (v/v) butanol (iii), 0% vs 4% (v/v) butanol; Benefit: dynamic gene expression profile governing cell response to increasing butanol concentrations | 32.4 | Stressor: 2%, 3%, 4% (v/v) of butanol; OD: mid-exèponential phase; Volume: 14 ml; Agitation: any; pH: n.a. | (Liu *et al.*, 2021) |
| *Synechocystis sp*. PCC 6803 | NO | iTRAQ-based LC–MS/MS | butanol stress response | Setup: identification of differentially expressed proteins comparing cells grown in medium supplemented with 0.20% (v/v) butanol vs control medium at (i) 24h and (ii) 48h; Benefit: proteomic analysis of gene expression programs modulated by exposure to butanol | 1.62 | Stressor: 0, 0.15%, 0.20%, 0.25% (v/v) butanol; OD: 0.5; Volume: 50 ml; Agitation: 130 rpm; pH: 7.5 | (Tian *et al.*, 2013) |
| *Sulfolobus acidocaldarius* DSM 639 | NO | RNA sequencing analysis, iTRAQ-based LC-MS/MS analysis | butanol stress response | Setup: Transcriptome analysis comparing the response of planktonic (PL) and biofilm (BF) cells in 0% and 0.5% or 1% (v/v) butanol; Proteome anaysis comparing the response of BF cells in 0% and 1% (v/v) butano Benefit: elucidation of the changes in transcript/protein levels in response to butanol challenge in liquid/biofilm cultures | 20.3 (BF cells) | *Biofilm culture*: Stressor: 0.5%, 1%, 1.5% (v/v) butanol; OD: ; Volume: 96-well polystyrene microtiter plate; Agitation: any; pH: 3. *Liquid culture*: Stressor: 0.5%, 1%, 1.5% (v/v) butanol; OD: 0.05; Volume: n.a.; Agitation: 180 rpm; pH: 3 | (Benninghoff *et al.*, 2021) |
| *Synechocystis sp*. PCC 6803 | NO | RNA sequencing analysis, GC-MS based metabolomics analysis | butanol stress response | Setup: transcriptomic and metabolomic analyses of butanol stress response; Benefit: identification of putative genetic programmes affected by butanol threat and validation by construction of knockout mutants | 1.62 | Stressor: 0.20% (v/v) butanol; OD: 0.5; Volume: 50 ml; Agitation: 130 rpm; pH: 7.5 | (Zhu *et al.*, 2013) |
| *E. coli* K-12 strain, BW25113 (Δ(*araD*-*araB*)567, Δ *lacZ4787*(::*rrnB*-3), *lambda-*, *rph-1*, Δ(*rhaD-rhaB*)568, *hsdR514*) | YES | array-CGH | butanol stress response | Setup: exposition of a genomic library covering *E. coli* to increasing butanol concentrations via batch serial transfers and analysis of enriched/depleted genes by the use of overexpression library/knockout collection; Benefit: identification of genes whose overexpression/deletion decreases the growth-inhibitory effects of butanol | 13.8 | Stressor: 0%, 0.9%, 1.3% and 1.7% (v/v) butanol; OD: 0.6; Volume: 25 ml; Agitation: n.a.; pH: n.a. | (Reyes *et al.*, 2011) |
| Several yeast *Saccharomyces cerevisiae* strains | YES | Analysis of ribosome distribution on sucrose gradients | butanol stress response | Setup: translational activity assay following butanol addition; Benefit: characterization of the translational control pathways affected by butanol challenge | 8.1 | Stressor: 1% (v/v) butanol; OD: 0.7; Volume: 15 ml; Agitation: n.a.; pH: n.a. | (Ashe *et al.*, 2001) |
| Several strains of genus *Candida*, *Escherichia*, *Lactobacillus*, *Pichia, Saccharomyces, Zymomonas* | NO | Any | butanol stress response | Setup: growth assay; Benefit: evaluation of the potential of alternative hosts for butanol production | strain-dependent | Stressor: 0, 1%, 2%, 2.5%, 3% (v/v) butanol; OD: 0.1–0.3; Volume: n.a.; Agitation: strain-dependent; pH: n.a. | (Knoshaug and Zhang, 2009) |
| *Bacillus subtilis* strain 168 | NO | LC-MS analysis of metabolome | butanol stress response | Setup: untargeted metabolomics coupled with quantitative real-time reverse transcriptase PCR and scanning electron microscopy to link the metabolic changes to the expression level of their likely related gene transcripts and to the cell morphology changes; Benefit: identification of changes in lipid metabolism and cell morphology upon butanol stress | 11.3 | Stressor: 0%, 0.6%, 0.8%, 1%, 1%, 1.4% (v/v) butanol; OD: 0.2–0.3; Volume: 20 ml; Agitation: any; pH: n.a. | (Vinayavekhin *et al.*, 2015) |
| *E. coli* mutant B8, *E. coli* wild-type | YES | cDNA microarray analysis | butanol stress tolerance | Setup: global transcriptional machinery engineering followed by differential transcriptome analysis between the isolated mutant and wild-type strain; Benefit: identification of tolerance-related genetic programmes | 16.2 | Stressor: 1.2% to 2.2% (v/v) butanol; OD: 0.2; Volume: 24-well plate; Agitation: 120 rpm; pH: n.a. | (Si *et al.*, 2016) |
| *E. coli* DH5α | YES | Any | butanol stress tolerance | Setup: random mutagenesis and enrichment screening to isolate butanol tolerant gene variants followed by mutant selection by error-prone PCR libraries and DNA shuffling library construction to generate the final butanol tolerant mutant; Benefit: generation of a strain endowed with improved butanol tolerance by means of transcription factor engineering | 16.2 | Stressor: 0%, 0.8%, 1.2%, 2% (v/v) butanol; OD: 0.6; Volume: agar plate; Agitation: any; pH: n.a. | (Zhang *et al.*, 2012) |
| several *E. coli* strains | YES | Any | butanol stress tolerance | Setup: combinatorial engineering approach impinged on the concerted action of multiple cellular functions endowing cells with enhanced butanol tolerance; Benefit: optimization of strain butanol tolerance | 16.2 | Stressor: 0%, 1%, 1.5%, 2% (v/v) butanol; OD: 0.1; Volume: 30 ml; Agitation: 200 rpm; pH: n.a. | (Bui *et al.*, 2015) |
| *C. acetobutylicum* mutant strain Y217, *C. acetobutylicum* ATCC 824 | NO | genome-wide resequencing, SNPs and InDels analysis | butanol stress tolerance | Setup: characterization of irradiation-induced mutant endowed with enhanced butanol tolerance; Benefit: elucidation of the relationships between changes in cell membrane permeability, membrane potential, and cell membrane fatty acid content with butanol tolerance under butanol stress conditions | 16.2 | Stressor: 0%, 1%, 2.0%, 2.5%, and 3.0% (v/v) butanol; OD: 1; Volume: 50 ml; Agitation: any; pH: 6.5 | (Gao *et al.*, 2021) |
| *E. coli* JM109 | YES | Any | butanol stress tolerance | Setup: mining class-specific enzymes for effectiveness in enhancing butanol tolerance by overexpression followed by random mutagenesis of the selected enzyme and characterization thereof; Benefit: achievement of strain mutant able to tolerate increased butanol concentration | 8.1 | Stressor: 1% (v/v) butanol; OD: 0.7-0.8; Volume: 30 ml; Agitation: any; pH: 7 | (Xu *et al.*, 2019) |

***References***

Alsaker, K. V., Paredes, C., and Papoutsakis, E.T. (2010) Metabolite stress and tolerance in the production of biofuels and chemicals: Gene-expression-based systems analysis of butanol, butyrate, and acetate stresses in the anaerobe Clostridium acetobutylicum. *Biotechnol Bioeng* **105**: 1131–1147.

Alsaker, K. V., Spitzer, T.R., and Papoutsakis, E.T. (2004) Transcriptional Analysis of spo0A Overexpression in Clostridium acetobutylicum and Its Effect on the Cell’s Response to Butanol Stress. *J Bacteriol* **186**: 1959–1971.

Ashe, M.P., Slaven, J.W., de Long, S.K., Ibrahimo, S., and Sachs, A.B. (2001) A novel elF2B-dependent mechanism of translational control in yeast as a response to fusel alcohols. *EMBO J* **20**: 6464–6474.

Baer, S.H., Blaschek, H.P., and Smith, T.L. (1987) Effect of Butanol Challenge and Temperature on Lipid Composition and Membrane Fluidity of Butanol-Tolerant Clostridium acetobutylicum. *Appl Environ Microbiol* **53**: 2854–2861.

Bao, G., Dong, H., Zhu, Y., Mao, S., Zhang, T., Zhang, Y., et al. (2014) Comparative genomic and proteomic analyses of Clostridium acetobutylicum Rh8 and its parent strain DSM 1731 revealed new understandings on butanol tolerance. *Biochem Biophys Res Commun* **450**: 1612–1618.

Benninghoff, J.C., Kuschmierz, L., Zhou, X., Albersmeier, A., Pham, T.K., Busche, T., et al. (2021) Exposure to 1-Butanol Exemplifies the Response of the Thermoacidophilic Archaeon Sulfolobus acidocaldarius to Solvent Stress. *Appl Environ Microbiol* **87**: 1–19.

Borden, J.R. and Papoutsakis, E.T. (2007) Dynamics of genomic-library enrichment and identification of solvent tolerance genes for Clostridium acetobutylicum. *Appl Environ Microbiol* **73**: 3061–3068.

Bowles, L.K. and Ellefson, W.L. (1985) Effects of butanol on Clostridium acetobutylicum. *Appl Environ Microbiol* **50**: 1165–1170.

Bui, L.M., Lee, J.Y., Geraldi, A., Rahman, Z., Lee, J.H., and Kim, S.C. (2015) Improved n-butanol tolerance in Escherichia coli by controlling membrane related functions. *J Biotechnol* **204**: 33–44.

Gao, Y., Zhou, X., Zhang, M.M., Liu, Y.J., Guo, X.P., Lei, C.R., et al. (2021) Response characteristics of the membrane integrity and physiological activities of the mutant strain Y217 under exogenous butanol stress. *Appl Microbiol Biotechnol* **105**: 2455–2472.

Janssen, H., Grimmler, C., Ehrenreich, A., Bahl, H., and Fischer, R.J. (2012) A transcriptional study of acidogenic chemostat cells of Clostridium acetobutylicum-Solvent stress caused by a transient n-butanol pulse. *J Biotechnol* **161**: 354–365.

Jia, K., Zhang, Y., and Li, Y. (2012) Identification and characterization of two functionally unknown genes involved in butanol tolerance of clostridium acetobutylicum. *PLoS One* **7**:.

Knoshaug, E.P. and Zhang, M. (2009) Butanol tolerance in a selection of microorganisms. In *Applied Biochemistry and Biotechnology*. Appl Biochem Biotechnol, pp. 13–20.

Lin, Y.L. and Blaschek, H.P. (1983) Butanol production by a butanol-tolerant strain of Clostridium acetobutylicum in extruded corn broth. *Appl Environ Microbiol* **45**: 966–973.

Liu, S., Qureshi, N., Bischoff, K., and Darie, C.C. (2021) Proteomic Analysis Identifies Dysregulated Proteins in Butanol-Tolerant Gram-Positive Lactobacillus mucosae BR0713-33. *ACS Omega* **6**: 4034–4043.

Liu, X.B., Gu, Q.Y., and Yu, X. Bin (2013a) Repetitive domestication to enhance butanol tolerance and production in Clostridium acetobutylicum through artificial simulation of bio-evolution. *Bioresour Technol* **130**: 638–643.

Liu, X.B., Gu, Q.Y., and Yu, X. Bin (2013b) Repetitive domestication to enhance butanol tolerance and production in Clostridium acetobutylicum through artificial simulation of bio-evolution. *Bioresour Technol* **130**: 638–643.

Liu, X.B., Gu, Q.Y., Yu, X. Bin, and Luo, W. (2012) Enhancement of butanol tolerance and butanol yield in Clostridium acetobutylicum mutant NT642 obtained by nitrogen ion beam implantation. *J Microbiol* **50**: 1024–1028.

Mao, S., Luo, Y., Zhang, T., Li, J., Bao, G., Zhu, Y., et al. (2010) Proteome reference map and comparative proteomic analysis between a wild type clostridium acetobutylicumDSM 1731 and its mutant with enhanced butanol tolerance and butanol yield. *J Proteome Res* **9**: 3046–3061.

Petrov, K., Arsov, A., and Petrova, P. (2021) Butanol tolerance of Lactiplantibacillus plantarum: A transcriptome study. *Genes (Basel)* **12**: 1–21.

Reyes, L.H., Almario, M.P., and Kao, K.C. (2011) Genomic library screens for genes involved in n-butanol tolerance in Escherichia coli. *PLoS One* **6**:.

Rühl, J., Schmid, A., and Blank, L.M. (2009) Selected Pseudomonas putida strains able to grow in the presence of high butanol concentrations. *Appl Environ Microbiol* **75**: 4653–4656.

Rutherford, B.J., Dahl, R.H., Price, R.E., Szmidt, H.L., Benke, P.I., Mukhopadhyay, A., and Keasling, J.D. (2010) Functional genomic study of exogenous n-butanol stress in Escherichia coli. *Appl Environ Microbiol* **76**: 1935–1945.

Schwarz, K.M., Kuit, W., Grimmler, C., Ehrenreich, A., and Kengen, S.W.M. (2012) A transcriptional study of acidogenic chemostat cells of Clostridium acetobutylicum - Cellular behavior in adaptation to n-butanol. *J Biotechnol* **161**: 366–377.

Si, H.M., Zhang, F., Wu, A.N., Han, R.Z., Xu, G.C., and Ni, Y. (2016) DNA microarray of global transcription factor mutant reveals membrane-related proteins involved in n-butanol tolerance in Escherichia coli. *Biotechnol Biofuels* **9**:.

Tian, X., Chen, L., Wang, J., Qiao, J., and Zhang, W. (2013) Quantitative proteomics reveals dynamic responses of Synechocystis sp. PCC 6803 to next-generation biofuel butanol. *J Proteomics* **78**: 326–345.

Tomas, C.A., Beamish, J., and Papoutsakis, E.T. (2004) Transcriptional Analysis of Butanol Stress and Tolerance in Clostridium acetobutylicum. *J Bacteriol* **186**: 2006–2018.

Tomas, C.A., Welker, N.E., and Papoutsakis, E.T. (2003) Overexpression of groESL in Clostridium acetobutylicum results in increased solvent production and tolerance, prolonged metabolism, and changes in the cell’s transcriptional program. *Appl Environ Microbiol* **69**: 4951–4965.

Vasylkivska, M., Branska, B., Sedlar, K., Jureckova, K., Provaznik, I., and Patakova, P. (2020) Phenotypic and Genomic Analysis of Clostridium beijerinckii NRRL B-598 Mutants With Increased Butanol Tolerance. *Front Bioeng Biotechnol* **8**: 1307.

Venkataramanan, K.P., Jones, S.W., McCormick, K.P., Kunjeti, S.G., Ralston, M.T., Meyers, B.C., and Papoutsakis, E.T. (2013) The Clostridium small RNome that responds to stress: The paradigm and importance of toxic metabolite stress in C. acetobutylicum. *BMC Genomics* **14**:.

Venkataramanan, K.P., Min, L., Hou, S., Jones, S.W., Ralston, M.T., Lee, K.H., and Papoutsakis, E.T. (2015) Complex and extensive post-transcriptional regulation revealed by integrative proteomic and transcriptomic analysis of metabolite stress response in Clostridium acetobutylicum. *Biotechnol Biofuels* **8**:.

Vinayavekhin, N., Mahipant, G., Vangnai, A.S., and Sangvanich, P. (2015) Untargeted metabolomics analysis revealed changes in the composition of glycerolipids and phospholipids in Bacillus subtilis under 1-butanol stress. *Appl Microbiol Biotechnol* **99**: 5971–5983.

Vollherbst Schneck, K., Sands, J.A., and Montenecourt, B.S. (1984) Effect of butanol on lipid composition and fluidity of Clostridium acetobutylicum ATCC 824. *Appl Environ Microbiol* **47**: 193–194.

Wang, Q., Venkataramanan, K.P., Huang, H., Papoutsakis, E.T., and Wu, C.H. (2013) Transcription factors and genetic circuits orchestrating the complex, multilayered response of Clostridium acetobutylicum to butanol and butyrate stress. *BMC Syst Biol* **7**: 1–17.

Wang, Y.F., Tian, J., Ji, Z.H., Song, M.Y., and Li, H. (2016) Intracellular metabolic changes of Clostridium acetobutylicum and promotion to butanol tolerance during biobutanol fermentation. *Int J Biochem Cell Biol* **78**: 297–306.

Wen, Z., Ledesma-Amaro, R., Lin, J., Jiang, Y., and Yangd, S. (2019) Improved n-butanol production from clostridium cellulovorans by integrated metabolic and evolutionary engineering. *Appl Environ Microbiol* **85**:.

Winkler, J. and Kao, K.C. (2011) Transcriptional analysis of Lactobacillus brevis to N-butanol and ferulic acid stress responses. *PLoS One* **6**:.

Xu, G., Wu, A., Xiao, L., Han, R., and Ni, Y. (2019) Enhancing butanol tolerance of Escherichia coli reveals hydrophobic interaction of multi-tasking chaperone SecB. *Biotechnol Biofuels* **12**:.

Xu, M., Zhao, J., Yu, L., Tang, I.C., Xue, C., and Yang, S.T. (2015) Engineering Clostridium acetobutylicum with a histidine kinase knockout for enhanced n-butanol tolerance and production. *Appl Microbiol Biotechnol* **99**: 1011–1022.

Xu, M., Zhao, J., Yu, L., and Yang, S.T. (2017) Comparative genomic analysis of Clostridium acetobutylicum for understanding the mutations contributing to enhanced butanol tolerance and production. *J Biotechnol* **263**: 36–44.

Yang, S.-T. and Zhao, J. (2011) Adaptive engineering of clostridium for increased butanol production.

Yang, X., Xu, M., and Yang, S.T. (2015) Metabolic and process engineering of Clostridium cellulovorans for biofuel production from cellulose. *Metab Eng* **32**: 39–48.

Zhang, H., Chong, H., Ching, C.B., Song, H., and Jiang, R. (2012) Engineering global transcription factor cyclic AMP receptor protein of Escherichia coli for improved 1-butanol tolerance. *Appl Microbiol Biotechnol* **94**: 1107–1117.

Zhu, H., Ren, X., Wang, J., Song, Z., Shi, M., Qiao, J., et al. (2013) Integrated OMICS guided engineering of biofuel butanol-tolerance in photosynthetic Synechocystis sp. PCC 6803. *Biotechnol Biofuels* **6**:.
